# Supramolecular Peptide Nanofiber Hydrogels for Bone Tissue Engineering: From Multihierarchical Fabrications to Comprehensive Applications

**DOI:** 10.1002/advs.202103820

**Published:** 2022-02-07

**Authors:** Zhuowen Hao, Hanke Li, Yi Wang, Yingkun Hu, Tianhong Chen, Shuwei Zhang, Xiaodong Guo, Lin Cai, Jingfeng Li

**Affiliations:** ^1^ Department of Orthopedics Zhongnan Hospital of Wuhan University Donghu Road 169 Wuhan 430071 China; ^2^ Department of Orthopedics Union Hospital Tongji Medical College Huazhong University of Science and Technology Jiefang Road 1277 Wuhan 430022 China

**Keywords:** applications, bone regeneration, fabrications, nanofiber hydrogels, supramolecular peptides

## Abstract

Bone tissue engineering is becoming an ideal strategy to replace autologous bone grafts for surgical bone repair, but the multihierarchical complexity of natural bone is still difficult to emulate due to the lack of suitable biomaterials. Supramolecular peptide nanofiber hydrogels (SPNHs) are emerging biomaterials because of their inherent biocompatibility, satisfied biodegradability, high purity, facile functionalization, and tunable mechanical properties. This review initially focuses on the multihierarchical fabrications by SPNHs to emulate natural bony extracellular matrix. Structurally, supramolecular peptides based on distinctive building blocks can assemble into nanofiber hydrogels, which can be used as nanomorphology‐mimetic scaffolds for tissue engineering. Biochemically, bioactive motifs and bioactive factors can be covalently tethered or physically absorbed to SPNHs to endow various functions depending on physiological and pharmacological requirements. Mechanically, four strategies are summarized to optimize the biophysical microenvironment of SPNHs for bone regeneration. Furthermore, comprehensive applications about SPNHs for bone tissue engineering are reviewed. The biomaterials can be directly used in the form of injectable hydrogels or composite nanoscaffolds, or they can be used to construct engineered bone grafts by bioprinting or bioreactors. Finally, continuing challenges and outlook are discussed.

## Graphical abstract

This review proposes a novel concept to construct bone repair biomaterials based on supramolecular peptide nanofiber hydrogels, namely multihierarchical fabrications by structural design, biochemical functionalization, and mechanical optimization. Then, it discusses the comprehensive applications of supramolecular peptide nanofiber hydrogels for bone tissue engineering, i.e., from injectable hydrogels or composite nanoscaffolds to bioprinting or bioreactors to construct engineered bone grafts.

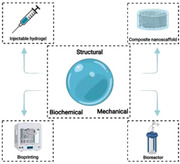

## 1 Introduction

Bone is a highly mineralized and neurovascularized hierarchical tissue that shows potential of self‐healing after injuries. However, clinic failure on bone fracture healing may vary from 5% to 10%, which is correlated with various risk factors, including serious injury, diabetes, osteoporosis, obesity, and smoking.^[^ ^1^ ^]^ Besides, tumor ablation, infection, and other orthopedic diseases may cause critical‐sized bone defects with microenvironment unsuitable for bone regeneration. Bone delayed union or nonunion may result in great losses for individuals and the society.

Autologous bone grafting is presently the gold standard for surgical bone repair, because allografts provide osteoinductive signals, osteogenic cells, and an osteoconductive matrix.^[^ ^2^ ^]^ However, this procedure shows some disadvantages, including limited availability, variable resorption, increased morbidity, and additional surgery.^[^ ^3^ ^]^ Allografts and xenografts are widely available, and they do not cause donor site morbidity; however, they are highly related to the risks of immune rejection and pathogen transmission.^[^ ^4^ ^]^ Therefore, bone tissue engineering shows promising prospect for surgical bone repair, with four pillars of biomaterial scaffolds, seed cells, bioactive factors, and biophysical stimuli.^[^ ^5^ ^]^ However, what consistently impedes its clinic application is the lack of suitable biomaterials that could rationally integrate the other three key components. Hydrogels may be ideal scaffolds for bone tissue engineering because they structurally resemble the natural extracellular matrix (ECM), which could support cell behavior, including migration, adhesion, proliferation, and differentiation.^[^ ^6^ ^]^ Biochemically, bioactive motifs could be covalently tethered to the network of hydrogel to direct cell behavior or fate, and bioactive factors could be physically adsorbed in hydrogel matrix to be released in a sustained manner, which depends on cross‐link density, matrix affinity to factors, and biomaterial degradation rate.^[^ ^7^ ^]^ Even their mechanical properties could also be easily adjusted to provide biophysical stimuli for tissue engineering.^[^ ^8^ ^]^

Hydrogelating biomaterials could be categorized into natural biomaterials and synthetic biomaterials (synthetic polymers and supramolecular peptides). Natural biomaterials (such as alginate,^[^ ^9^ ^]^ collagen,^[^ ^10^ ^]^ chitosan,^[^ ^11^ ^]^ etc.) are limited because they may cause pathogen transmission and immune rejection and due to unexpected growth factors that may disturb bone regeneration with product quality varying from batch to batch. For hydrogelating synthetic polymers, such as poly(ethylene glycol) (PEG),^[^ ^12^ ^]^ poly(*N*‐isopropylacrylamide),^[^ ^13^ ^]^ and poly(lactide‐*co*‐glycolide),^[^ ^14^ ^]^ some harmful chemical substances possibly reside in the substrates, and the introduction of bioactive motifs is complex and intricate. Therefore, hydrogelating supramolecular peptides are emerging biomaterials for tissue engineering because of their inherent biocompatibility, satisfied biodegradability, high purity, facile functionalization, and tunable mechanical properties. In an aqueous solution with specific conditions (such as pH, ion strength, temperature, or mental ions), supramolecular peptides based on distinctive building blocks first assemble into nanofibers, which aggregate into nanofibril networks, namely, supramolecular peptide nanofiber hydrogels (SPNHs).^[^ ^15^ ^]^ These SPNHs have been widely used in various biomedical applications, including cell culture, drug delivery, tumor therapy, antimicrobial materials, tissue engineering, and bioprinting.^[^ ^16^ ^]^

In the field of bone tissue engineering, biomaterials that are currently used as bone substitutes in clinical trials mainly include undegradable metallic implants such as printed titanium implants (NCT03941028), degradable bioceramics such as bioactive glass (NCT04767243), and degradable polymers such as hyaluronic acid (HA) (NCT05073575). However, current bone substitutes under clinic trials fail to meet the requirement for bone regeneration by abundant biochemical functions, including basic biochemical functions (cell adhesion, cell recruitment, and matrix degradation), improved biochemical functions (matrix biomineralization, osteogenesis, angiogenesis, neurogenesis, and immune regulation), and additional biochemical functions (sterilization and tumor suppression). These biochemical functions are mainly fulfilled by the introduction of bioactive motifs such as cell adhesion peptides (CAPs), or bioactive factors such as bone morphogenetic proteins (BMPs). Compared with biomaterials currently in clinical trials, SPNHs could be facilely endowed with multiple biochemical functions by conjugating bioactive motifs and absorbing bioactive factors. Besides, other advantages of SPNHs over current bone substitutes include: 1) SPNHs show artificial microenvironment analogous to native ECM, which could modulate cells by ECM–cell interactions; 2) SPNHs almost initiate no adverse immune response or tissue inflammation when implanted into the body; 3) there is no toxic substance during degeneration because they are mainly constructed by amino acids; and 4) SPNHs can be synthesized in high purity without adverse substances and batch differences. Therefore, SPNHs are emerging biomaterials for bone tissue engineering which should be multihierarchically fabricated and comprehensively applied.

**Figure 1:**
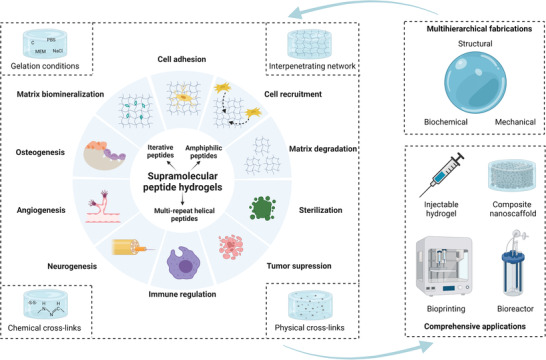
Overview of supramolecular peptide nanofiber hydrogels for bone tissue engineering. Created with BioRender.com.

Currently, commercial SPNH products that have been used for clinical trials mainly include PuraStat and T45K for hemostasis,^[^ ^17^ ^]^ P11‐4 for remineralization,^[^ ^18^ ^]^ as well as Sciobio, Saienbei, Nafumei, Nafujia, Nafubao, and Nafubang which are used as wound liquid dressing.^[^ ^19^ ^]^ Although some reviews on SPNHs for tissue engineering have been published,^[^ ^20^ ^]^ the present review helps provide multihierarchical fabrication strategies of SPNHs, especially for bone tissue engineering and then their comprehensive applications (**Figure** **1**). Herein, for structural design, various hydrogelating supramolecular peptides that could assemble into SPNHs analogous to natural ECM were summarized on the basis of distinctive building blocks. For biochemical functionalization, two strategies (covalent conjugation of bioactive motifs and physical absorption of bioactive factors) were presented to endow basic, improved, and additional functions for bone regeneration. For mechanical properties, four methodologies were reviewed to optimize the biophysical microenvironment of SPNHs, namely, to improve the stiffness or elasticity. Furthermore, the comprehensive applications of SPNHs for bone tissue engineering were summarized. Not only can they be used in the form of injectable hydrogels or composite nanoscaffolds, but also they can be also utilized to construct engineered bone grafts by bioprinting or bioreactors. This review aimed to successfully fulfill the clinical application of SPNHs for surgical bone repair.

## 2 Structural Design as ECM‐Like Scaffolds

**Figure 2:**
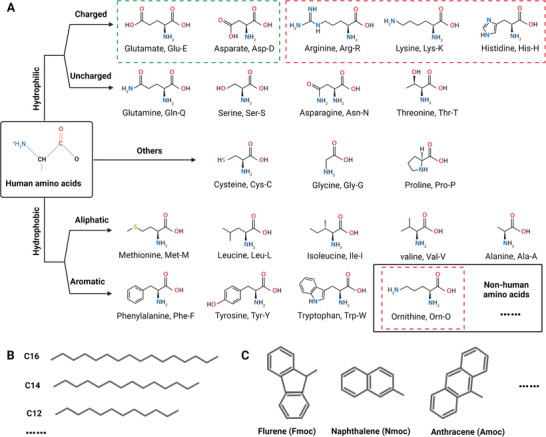
A–C) Basic structures of building components for supramolecular peptides. (A) Amino acid structures and classification, including human amino acids and nonhuman amino acids. Reproduced with permission.^[^ ^21^ ^]^ Copyright 2017, Elsevier. (B) Structures of hydrophobic aliphatic alkyl chains with distinctive number of carbons. (C) Structures of some N‐terminal aromatic groups. Created with BioRender.com.

Supramolecular peptides are mainly constructed by rational arrangements of amino acids (**Figure** **2**A). Human amino acids could be divided into hydrophilic amino acids (charged and uncharged) and hydrophobic amino acids (aliphatic and aromatic). Besides, some specific amino acids could be separated into one group because of their specific functions during peptide design.^[^ ^21^ ^]^ For example, glycine could be used as a linker to flexibly expose bioactive motifs outside the backbone of supramolecular peptides. Cysteine could be oxidized to form disulfide bonds for intermolecular cross‐linking or the introduction of bioactive motifs, and proline could be used to construct *β*‐turn structure due to its special structure. Nonhuman amino acids could also be used for the construction of supramolecular peptides. For example, ornithine could be used a supplement for positive‐charged group^[^ ^22^ ^]^ and dehydrophenylalanine for aromatic group.^[^ ^23^ ^]^ d‐amino acids could be used to construct chiral supramolecular peptides, whose antiproteinase stability are enhanced.^[^ ^24^ ^]^ Moreover, nonamino acid components, such as hydrophobic aliphatic alkyl chains (Figure 2B) and aromatic epitopes (Figure 2C) that provide *π*–*π* stacking interactions, could be used for the design of supramolecular peptides.

The assembly of supramolecular peptides is mainly based on intramolecular or intermolecular noncovalent interactions, including hydrogen bonds, halogen bond, van der Waals forces, *π*–*π* stacking interactions, electrostatic interactions, and hydrophilic and hydrophobic interactions.^[^ ^20^, ^25^ ^]^ Under certain conditions, well‐designed supramolecular peptides based on distinctive building blocks assemble into nanofibers with different structures, including *α*‐helix, *β*‐sheet, *β*‐turn, *β*‐spiral, and micelle, which then cross‐link into ECM‐like hydrogels. Therefore, the assembling physical properties endow supramolecular peptides with the ability to form SPNHs, which could be used not only as nanomatrix for 2D surface culture or 3D encapsulation culture in vitro to explore the interaction between ECM and stem cells, but also as implantable grafts for the treatment of bone defects in vivo.

As emerging biomaterials, SPNHs have been used in the clinic for hemostasis, wound healing, and the remineralization of white spot lesions,^[^ ^19^ ^]^ but they have not been used for clinic bone repair. The critical points for the translation of SPNHs to clinical products for bone repair are reasonable functionalization and mechanical optimization, which could endow SPNHs a multifunctional microenvironment for bone regeneration and improve the poor mechanical properties (0.01–1 kPa) caused by noncovalent interactions.^[^ ^26^ ^]^ The subsequent biochemical and mechanical fabrications are mainly based on the primary structural design. So, the structural design is the footstone for clinic translation as well as multihierarchical fabrications. In accordance with their design principles, SPNHs were categorized into three types: iterative peptides, amphiphilic peptides, and multirepeat helical peptides.

### 2.1 Iterative Peptides

Iterative peptides refer to those with iterative residues (namely, alternative hydrophilic and hydrophobic amino acids), which could assemble into amyloid‐like nanofibers on the basis of *β*‐sheet structure. In accordance with the choice and distribution of hydrophilic amino acids, they could be divided into five distinctive peptides: ionic‐complementary peptides, self‐repulsive peptides, multidomain peptides, *β*‐hairpin peptides, and *β*‐tape peptides.

#### 2.1.1 Ionic‐Complementary Peptides

**Figure 3:**
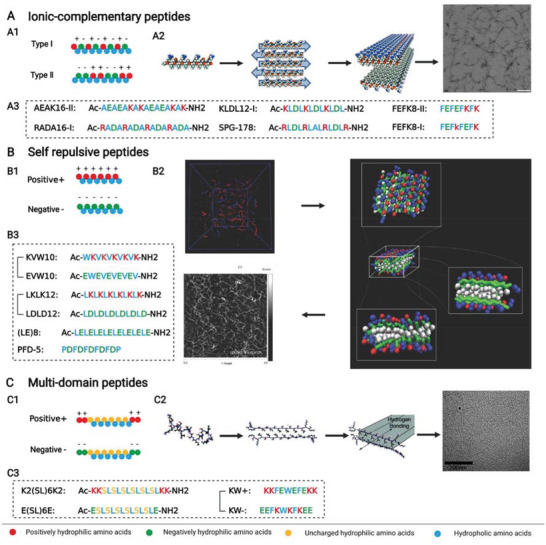
A–C) Ionic‐complementary peptides, self‐repulsive peptides, and multi‐domain peptides from iterative peptides. A1) Design principle of ionic‐complementary peptides. A2) Structure of SPG‐178 peptide coupled with its assembly to fibers and nanofiber scaffold (transmission electron microscopic image). Reproduced with permission.^[^ ^49^ ^]^ Copyright 2012, Elsevier. A3) Some peptide sequences and structures of ionic‐complementary peptides. B1) Design principle of self‐repulsive peptides. B2) Structure of LKLK12 and LDLD12 peptides coupled with their assembly to fibers and nanofiber scaffold (atomic force image). Reproduced with permission.^[^ ^56^ ^]^ Copyright 2014, Wiley‐VCH. B3) Some peptide sequences and structures of self‐repulsive peptides. C1) Design principle of multidomain peptides. C2) Structure of K_2_(SL)_6_K_2_ peptide coupled with its assembly to fibers and nanofiber scaffold (transmission electron microscopic image). Reproduced with permission.^[^ ^57^ ^]^ Copyright 2009, American Chemical Society. C3) Some peptide sequences and structures of multidomain peptides. Created with BioRender.com.

Ionic‐complementary peptides are the most widely used iterative peptides, whose hydrophilic amino acids consist of positive and negative groups (**Figure** **3**A). In accordance with the arrangement of charged amino acids, ionic‐complementary peptides could be divided into different types, such as type I (− + − + − + − +) and type II (− − + + − − + +).^[^ ^27^ ^]^ In aqueous solutions, hydrogen bonding and electrostatic interaction mainly induce their assembly. Hydrophobic residues tend to form inner hydrophobic layers, while charged amino acids tend to form outer hydrophilic layers. Then, two hydrophobic layers close to each other and form sandwich‐like structures, which may further slide into nanofibers.^[^ ^28^ ^]^ EAK16‐II (CH_3_CO—AEAEAKAKAEAEAKAK—NH_2_) is the first well‐established ionic‐complementary peptide derived from a Z‐DNA binding protein of yeast named Zoutin.^[^ ^29^ ^]^ In salt solutions, the peptide spontaneously self‐assembles into *β*‐sheet nanofibers and further forms a macroscopic matrix.^[^ ^30^ ^]^ A research team used arginine to substitute lysine and aspartate to glutamate to further simulate arginine‐glycine‐asparate (RGD) or arginine‐glycine‐asparate‐serine (RGDS) which are common CAPs, thus designing RADA16‐I (CH_3_CO—RADARADARADARADA—NH_2_) which is generally called RADA16 and RADA16‐II (CH_3_CO—RARADADARARADADA—NH_2_).^[^ ^31^ ^]^ EAK16‐II, RADA16‐I, and RADA16‐II could form *β*‐sheet nanofiber hydrogels under physical conditions without discoverable immune response in vivo.^[^ ^31^, ^32^ ^]^ And RADA16‐II nanofiber hydrogel has been verified to mediate vascular endothelial cells to form capillary networks, illustrating that these peptides have potential for the regeneration of vascularized tissues such as bone.^[^ ^33^ ^]^ Considering that RADA16‐I contains four RAD motifs, showing comparable cell attachment activity with RGD,^[^ ^34^ ^]^ it has been widely used for bone tissue engineering. Hamada et al.^[^ ^35^ ^]^ used the RADA16‐I hydrogel to 3D culture mesenchymal stem cells (MSCs) under osteogenic induction medium, and found that seeded MSCs could be induced to osteogenically differentiate and show high alkaline phosphatase (ALP) activity and osteocalcin contents, which ultimately generate a 3D mineralized matrix. Misawa et al.^[^ ^36^ ^]^ implanted the RADA16‐I hydrogel or Matrigel to mouse calvaria bone defects, and found that bone defects treated by RADA16‐I hydrogel showed higher expression of osteogenic genes, more bone neoformation, and higher bone strength than those treated with Matrigel. In the ilium bone defect model of New Zealand rabbits, it was reported that the RADA16‐I hydrogel stimulated bone regeneration without adverse inflammation, while bone wax inhibited bone formation.^[^ ^37^ ^]^ Furthermore, it could also be utilized as a suitable carrier to deliver seed cells, such as bone marrow MSCs,^[^ ^38^ ^]^ induced pluripotent stem cells,^[^ ^39^ ^]^ adipose‐derived stem cells (ADSCs),^[^ ^40^ ^]^ dental pulp stem cells (DPSCs),^[^ ^41^ ^]^ and vascular endothelial cells (VECs)^[^ ^42^ ^]^ for bone repair. The above results showed that the RADA16‐I hydrogel is an attractive nanoscaffold for bone tissue engineering.

When alanine is substituted by more hydrophobic residue, such as leucine, isoleucine, phenylalanine, and tyrosine, the assembling tendency and mechanical strength will be enhanced.^[^ ^27^, ^43^ ^]^ So, the peptide length can be relatively reduced, which facilitates mass synthesis with low cost. KLDL12‐I (CH_3_CO—KLDLKLDLKLDL—NH_2_), usually called KLD‐12, is an ionic‐complementary peptide containing 12 residues which has been widely used in tissue engineering.^[^ ^44^ ^]^ Moreover, the application of aromatic residues which are more hydrophobic could further reduce peptide length. FEFEFKFK, FEFKFEFK, and E1Y9 (CH_3_CO—EYEYKYEYKY—NH_2_) are canonical examples that could assemble into nanofiber hydrogels.^[^ ^45^ ^]^ And the FEFKFEFK hydrogel has been used to culture MSCs under 3D osteogenic conditions, and the results showed that encapsuled MSCs could be induced to undergo osteogenic differentiation and secrete osteogenic markers such as type I collagen, ALP, and osteocalcin, and that mineralization could be induced within the hydrogel matrix.^[^ ^46^ ^]^ So, the FEFEFKFK hydrogel may be used as a preferable substitute to the RADA16‐I hydrogel, which shows several advantages: 1) shorter peptide sequence is more facile to produce with low cost; 2) improved hydrophobicity and *π*–*π* stacking among aromatic residues could facilitate hydrogelation and improve mechanical properties.^[^ ^47^ ^]^

In the last 10 years, some charged hydrophilic amino acids were replaced by uncharged hydrophobic amino acids to fabricate novel ionic‐complementary peptides that do not comply with the traditional design of ionic‐complementary peptides. Some examples include FEFOFK (H—FEFQFK—NH_2_)^[^ ^48^ ^]^ and SPG‐178 (CH_3_CO—RLDLRLALRLDLR—NH_2_).^[^ ^49^ ^]^ Compared with RADA16‐I, the isoelectric point of SPG‐178 was substantially increased from 6.1 to 11.5; thus, the SPG‐178 hydrogel was more stable and biocompatible.^[^ ^49^ ^]^ Tsukamoto et al.^[^ ^50^ ^]^ used the SPG‐178 hydrogel to 3D culture DPSCs under osteogenic induction medium, and found that the hydrogel supported DPSCs to express osteogenic genes and induce matrix calcium deposition in vitro and promoted bone neoformation in rat critical calvarial bone defects. Besides, SPG‐178 hydrogel was also implanted to rabbit posterolateral lumbar fusion and tibial intramedullary models, and more bone regeneration was also observed than other implants such as *β*‐tricalcium phosphate (*β*‐TCP) and bone chips.^[^ ^51^ ^]^ Therefore, these findings show that the SPG‐178 hydrogel may be an ideal biomaterial for clinical translation to treat critical bone defects.

#### 2.1.2 Self‐Repulsive Peptides

Self‐repulsive peptides are similar to ionic‐complementary peptides, but their hydrophilic amino acids are completely composed of negative amino acids or positive amino acids (Figure 3B). Self‐repulsive peptides could induce gelation by changing pH or ionic strength or blending with oppositely charged modules.^[^ ^52^ ^]^ In aqueous solutions, self‐repulsive peptides could also assemble into double layers of sandwich‐like *β*‐sheet nanofibers, which are reminiscent of those of ionic‐complementary peptides.^[^ ^53^ ^]^ When hydrogels are obtained by adjusting pH or the ionic strength, oppositely charged ions are needed to screen the resistance among peptides and exert as cross‐linkers. Compared with positive self‐repulsive peptides, negative modules, such as (LE)8 (CH_3_CO—LELELELELELELELE—NH_2_)^[^ ^53^ ^]^ and PFD‐5 (PDFDFDFDFDP),^[^ ^54^ ^]^ may be promising for bone tissue engineering because abundant carboxyl group could efficaciously induce biomineralization. Meanwhile, when hydrogels are formed by coassembly of two complementary self‐repulsive peptides, the initial pH and ionic strength may be preserved without microenvironment change; thus, seed cells or bioactive factors could be directly loaded to peptide storage solutions under physiological conditions, making coassembling self‐repulsive SPNHs more biocompatible than stimuli‐triggered SPNHs.^[^ ^55^ ^]^ On the basis of KLDL12‐I, Raspa et al.^[^ ^56^ ^]^ designed a pair of self‐repulsive peptides, LKLK12 (CH_3_CO—LKLKLKLKLKLK—NH_2_) and LDLD12 (CH_3_CO—LDLDLDLDLD—NH_2_). Then, a function motif (KLPGWSG) that binds to neural stem cells (NSCs) and determine their fate commitment was introduced to LDLD12, which was subsequently blended with LKLK12 to obtain a coassembling hydrogel. The function of this motif was retained after blending because the assembling was induced by LDLD12 and LKLK12, while the functional motif was exposed outside the assembling nanofibers.^[^ ^56^ ^]^ And the results revealed that the functional hydrogel containing KLPGWSG maintained the bioactivity of NSCs and induced their neurogenic differentiation in vitro, and promoted nerve regeneration when implanted to complete spinal cord transections in vivo.^[^ ^56^ ^]^ Considering that neurogenesis is highly needed during bone regeneration, the coassembling SPNH also shows great potential to be used in bone regenerative microenvironment for bone tissue engineering.

#### 2.1.3 Multidomain Peptides

Multidomain peptides are another iterative peptide peculiar to ABA structure (Figure 3C). The middle section (B) is composed of alternative hydrophobic and hydrophilic amino acids, while both flanking sections (A) comprise charged amino acids.^[^ ^57^ ^]^ Stimulated by oppositely charged multivalent ions, multidomain peptides may assemble into double layers of sandwich‐like *β*‐sheet nanofibers.^[^ ^58^ ^]^ Among many multidomain peptides, K_2_(SL)_6_K_2_ (CH_3_CO—KKSLSLSLSLSLSLKK—NH_2_) is preferable because of its inherent abilities of angiogenesis and neurogenesis without cells, proteins, or proteins, which can be attributed to that the SPNH initiates an inflammatory response and promotes the rapid endogenous cell infiltration, and then these infiltrated cells secrete a plethora of growth factors and cytokines to further stimulate cell recruitment, vascularization, neurogenesis, and scaffold remodeling.^[^ ^59^ ^]^ Although the K_2_(SL)_6_K_2_ hydrogel has not been used for bone regeneration, it may be an ideal scaffold for bone tissue engineering because of its inherent ability to induce angiogenesis and neurogenesis. Some researchers have designed a pair of mutually attractive multidomain peptides, KW+ (KKFEWEFEKK) and KW− (EEFKWKFKEE) to coassemble into nanofiber hydrogel after blending, which could avoid changes to pH or temperature during gelation that may adversely impact cell behavior.^[^ ^60^ ^]^ So, it is highly recommended to construct coassembling SPNHs for bone tissue engineering, and further studies are needed to explore their potential to be used as biomaterial scaffolds.

#### 2.1.4 *β*‐Hairpin Peptides

**Figure 4:**
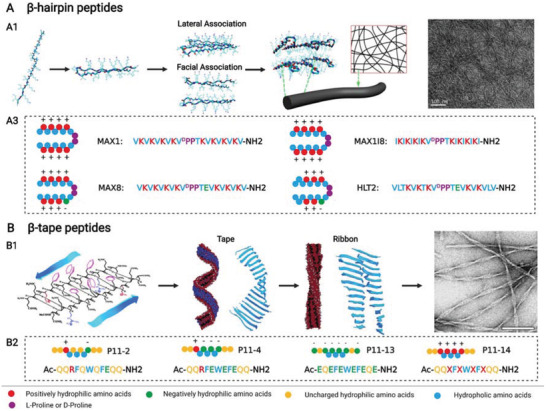
A,B) *β*‐Hairpin peptides and *β*‐tape peptides from iterative peptides. A1) Structure of MAX1I8 peptide coupled with its assembly to fibers and nanofiber scaffold (transmission electron microscopic image). Reproduced with permission.^[^ ^61^ ^]^ Copyright 2014, American Chemical Society. A2) Some peptide sequences and structures of *β*‐hairpin peptides. B1) Structure of P11‐2 peptide coupled with its assembly to fibers and nanofiber scaffold (transmission electron microscopic image). Reproduced with permission.^[^ ^66a^ ^]^ Copyright 2003, American Chemical Society. B2) Some peptide sequences and structures of *β*‐tape peptides. Created with BioRender.com.

*β*‐Hairpin peptides are distinctive from the above iterative peptides, and they are composed of two *β*‐sheet forming components and one *β*‐turn forming zone (**Figure** **4**A). The peptides could be assumed as rational insertion of the *β*‐turn forming sequence (such as V^D^PPT) into self‐repulsive peptides. In aqueous solutions, *β*‐hairpin peptides exist in random coils, which are folded when induced by exogenous stimuli. The folded *β*‐hairpin peptides assemble by lateral association and facial association into double layers of nanofibers, which ultimately cross‐link into stable hydrogels.^[^ ^61^ ^]^ MAX1 (VKVKVKVKV^D^PPTKVKVKVKV—NH_2_) is a parent peptide that could form nanofiber hydrogel induced by Dulbecco's modified Eagle medium.^[^ ^62^ ^]^ However, the major issue of MAX1 is its low gelation rate under neutral conditions, which causes heterogenous cellular distribution. For improvement of gelation rate and cellular encapsulation, more hydrophobic isoleucine residues which show stronger propensity for *β*‐sheet hydrogen bonding were used to substitute the valine residues of MAX1 to fabricate a novel *β*‐hairpin peptide called MAX1I8 (IKIKIKIKV^D^PPTKIKIKIKI—NH_2_).^[^ ^61^ ^]^ It was found that the MAX1I8 could assemble into hydrogel within 5 min, while the MAX1 failed to form hydrogel even after 30 min, so the MAX1I8 hydrogel could support homogeneous cell encapsulation.^[^ ^61^ ^]^ Changing charge distribution is another strategy to optimize MAX1. MAX8 (VKVKVKVKV^D^PPTEVKVKVKV—NH_2_) was fabricated with a negative glutamate to substitute a positive lysine at position 15 of MAX1, and the results showed that about 30 min were required for MAX1 to assemble into complete *β*‐sheet structures under physiological conditions, while less than 1 min was needed for MAX8.^[^ ^63^ ^]^ For biocompatibility enhancement, HLT2 (VLTKVKTKV^D^PPTEVKVKVLV—NH_2_) was designed with low formal charge states (from +7 of MAX8 to +5).^[^ ^64^ ^]^ And it was revelated that the less electropositive HLT2 hydrogel provided a more conductive matrix for chondrocyte encapsulation, delivery, and phenotype maintenance, and it supported higher cell viability than MAX8 hydrogel.^[^ ^64^ ^]^ However, the charged residues of current *β*‐hairpin peptides are mainly positive lysine residues. Considering that negatively charged amino acids tend to biomineralization for bone regeneration, using negative residues to construct the backbone of *β*‐hairpin peptides show great promise to design new *β*‐hairpin peptides for bone tissue engineering.

#### 2.1.5 *β*‐Tape Peptides

*β*‐Tape peptides show ABA structure, but their assembling behavior is distinctly different from that of multidomain peptides (Figure 4B). The peptides are designed by three critical criteria:^[^ ^65^ ^]^ 1) cross‐strand attraction between side chains to ensure the requisite antiparallel tape‐like structures, which include hydrogen binding, electrostatic interaction, or hydrophobic effect; 2) lateral recognition between adjacent strands or monomers to render 1D assembly and obtain homogeneous coacervated *β*‐sheet structures; and 3) controlled solubility resulting from strong solvent adhesion to the surface of the tapes. In suitable solutions, the rationally designed *β*‐tape peptides may assemble into a helical ribbon with double layers of *β*‐tapes, which may twist together and form nanofiber hydrogel.^[^ ^66^ ^]^ Natural K24 (KLEALYVLGFFGFFTLGIMLSYIR), which exists in a transmembrane domain of IsK protein, formed a transparent hydrogel in moderately polar solution, such as methanol, on the basis of *β*‐sheet tape structure; therefore, K24 provided an inspiration to design the first artificial *β*‐tape peptide called DN1(CH_3_CO—QQRFQWQFEQQ—NH_2_, also named P11‐2), which could form *β*‐sheet polymer tapes in aqueous solutions and then cross‐link into a stable hydrogel.^[^ ^67^ ^]^ The middle section was fabricated by alternative hydrophilic and hydrophobic amino acids, which was enhanced by *π*–*π* stacking from Phe4, Trp6, and Phe8. Both sides were composed of two glutamine residues. On the basis of P11‐2, a family of P11 *β*‐tape peptides was established, among which negative P11‐4 (CH_3_CO—QQRFEWEFEQQ—NH_2_) showed great potential for bone regeneration owing to its ability of biomineralization.^[^ ^68^ ^]^ Saha et al.^[^ ^69^ ^]^ implanted P11‐4 hydrogels to rat calvaria defects and set unfilled defects as control, and the results showed that P11‐4 hydrogels at 4 weeks dramatically promote almost complete bone healing when compared with unfilled defects.^[^ ^69^ ^]^ Considering that P11‐4 hydrogel has been tested for dental enamel regeneration in clinic trials, it may be also an attractive bone substitutes for clinic bone repair. Furthermore, coassembling SPNHs can be constructed by mutually complementary *β*‐tape peptides. Kyle et al.^[^ ^70^ ^]^ fabricated a pair of coassembling peptides, P11‐13 (CH_3_CO—EQEFEWEFEQE—NH_2_) and P11‐14 (CH_3_CO—QQXFXWXFXQQ—NH_2_, X refers to ornithine), which could assemble into nanofiber hydrogel by equimolar mixing. But related applications of coassembling SPNHs for bone tissue engineering are highly needed to explore in future, and whether coassembling SPNHs are superior to negative P11‐4 hydrogel for bone regeneration is still unknown.

### 2.2 Amphiphilic Peptides

Amphiphilic peptides are mainly characterized by a long hydrophobic tail and a hydrophilic head. In accordance with the different compositions of hydrophobic tails, amphiphilic peptides could be catharized into four groups: peptide amphiphiles (PAs), aromatic peptide amphiphiles (APAs), ultrashort peptides, and aromatic short peptides.

#### 2.2.1 Peptide Amphiphiles

**Figure 5:**
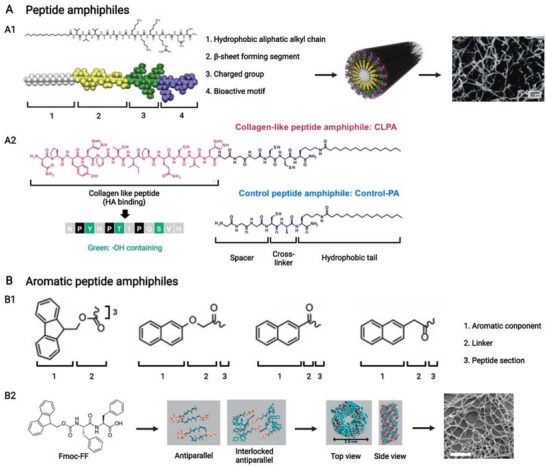
A,B) Peptide amphiphiles and aromatic peptide amphiphiles from amphiphilic peptides. A1) Canonical design principle of peptide amphiphile combined with its assembly to micelle (or nanofiber) and nanofiber scaffold (scanning electron microscopic image). Reproduced with permission.^[^ ^71^ ^]^ Copyright 2011, Elsevier. A2) Specific design principle of peptide amphiphile, that is, aliphatic alkyl chain is linked to the amino of side chain from positive residue but not to the amino of residue backbone. Reproduced with permission.^[^ ^79^ ^]^ Copyright 2015, American Chemical Society. B1) Design principle of aromatic peptide amphiphile combined with its assembly models. Reproduced with permission.^[^ ^80b^ ^]^ Copyright 2021, Wiley‐VCH. B2) Structure of Fmoc—FF peptide coupled with its assembly to fibers and nanofiber scaffold (scanning electron microscopic image). Reproduced with permission.^[^ ^81^ ^]^ Copyright 2008, Wiley‐VCH. Created with BioRender.com.

PAs are typically composed of four sections: 1) a hydrophobic aliphatic alkyl chain at N‐terminus, 2) a hydrophobic *β*‐sheet forming segment, 3) a charged group, and 4) a bioactive motif at C‐terminus (**Figure** **5**A1).^[^ ^71^ ^]^ The *β*‐sheet forming segment is used to stabilize the assembling micelle, and the charged group is used to improve the hydrophily of PAs. In suitable aqueous solutions, the hydrophobic aliphatic chains tend to gather inside, while the hydrophilic peptides tend to be exposed outside. Thus, cylindrical/fibril structures could be obtained, and they may further cross‐link into stable hydrogels triggered by polyvalent mental ions, such as Ca^2+^. Stupp and co‐workers^[^ ^72^ ^]^ established the first type of PA [CH_3_(CH_2_)_14_CONH—CCCCGGG—^P^SRGD] that could direct biomineralization after cross‐linking. Subsequently, the *β*‐sheet forming segment was optimized as hydrophobic amino acids for improved hydrophobicity, including AAAAGGG,^[^ ^73^ ^]^ AAALLL,^[^ ^74^ ^]^ and VVVAAA.^[^ ^75^ ^]^ For example, a SPNH assembled by CH_3_(CH_2_)_14_CONH—AAAAGGG—ERGD was used to culture MSCs in 3D conditions by normal medium or osteogenic induction medium, and the results showed that the SPNH promoted cell adhesion and proliferation when MSCs were cultured by normal medium and that the SPNH supported the osteogenic differentiation of MSCs when osteogenic induction medium was used.^[^ ^76^ ^]^ Mata et al.^[^ ^74^ ^]^ fabricated two oppositely charged peptides, CH_3_(CH_2_)_14_CONH—AAALLL—EE^P^SG and CH_3_(CH_2_)_14_CONH—AAALLL—KKRGDS, to coassemble into a SPNH. When implanted to critical femoral defects in rats, the SPNH that was coassembled with 95% CH_3_(CH_2_)_14_CONH—AAALLL—EE^P^SG and 5% CH_3_(CH_2_)_14_CONH—AAALLL—KKRGDS induced comparable bone regeneration with a clinically used allogenic grafts, but considerably higher bone formation than unphosphorylated hydrogel.^[^ ^74^ ^]^ Besides, highly hydrophilic bioactive motifs could be directly linked to the aliphatic alkyl chain without *β*‐sheet forming segment. A hydrophilic matrix metalloproteinase‐2 (MMP‐2) clearable sequence (GTAGLIGQ) coupled with a hydrophilic CAP (ERGDS) was used to construct a novel PA [CH_3_(CH_2_)_14_CONH—GTAGLIGQ—ERGDS], which may assemble into nanofiber hydrogel suitable for cell adhesion, infiltration, and proliferation.^[^ ^77^ ^]^

The hydrophobic aliphatic alkyl chain of some specific PAs is not linked to the side chain of peptides, but the amine of positive charged amino acids is.^[^ ^78^ ^]^ Jin et al.^[^ ^79^ ^]^ fabricated a new class of PA with four sections: 1) a bioactive motif at N‐terminus, 2) a spacer, 3) a cross‐linker, and 4) a hydrophobic aliphatic alkyl chain linked to the amine of lysine residue (Figure 5A2). And SPNHs could be obtained by coassembling bioactive PA and its control PA under pH 7.4 when the ratio of control PA was over 60%.^[^ ^79^ ^]^ But current studies have not utilized these specific PAs for bone tissue engineering, and the discrepancies between these specific PAs and cationic PAs are needed to explore.

#### 2.2.2 Aromatic Peptide Amphiphiles

APAs are generally composed of three sections: 1) an N‐terminal aromatic component, 2) a linker, and 3) a relatively hydrophilic peptide section (Figure 5B1).^[^ ^80^ ^]^ APAs are shorter than PAs, thereby allowing for a relative facile chemical synthesis. In aqueous solutions, APAs assemble by parallel, antiparallel, or interlocked antiparallel stacking arrangements to nanofibers, which subsequently cross‐link into hydrogels.^[^ ^80a^ ^]^ Highly aromatic epitope could be constructed by 9‐fluorenylmethoxycarbonyl (Fmoc), 9‐anthracenemethoxycarbonyl, and naphthalene‐2‐methoxycarbonyl,^[^ ^16a^ ^]^ among which the Fmoc group was widely used in tissue engineering. Fmoc—FF, a classic example of APAs, first assembled into an antiparallel structure, followed by interlocked antiparallel model, which ultimately formed cylindrical nanofiber (Figure 5B2).^[^ ^81^ ^]^ Wang et al.^[^ ^82^ ^]^ fabricated a SPNH by assembling Fmoc—FF and functional Fmoc—RGD, which supported the osteogenic differentiation of MSCs within hydrogel matrix. The researchers then implanted a graft fabricated by a gelatin sponge and the SPNH containing osteogenically induced MSCs to the dorsal skin of immunodeficient mice, and found that implanted sites show obvious calcium deposits, collagen synthesis, and ALP positive cells.^[^ ^82^ ^]^ So, Fmoc—FF/Fmoc—RGD coassembling SPNH may be an attractive scaffold for bone tissue engineering.^[^ ^82^ ^]^

#### 2.2.3 Ultrashort Peptides

**Figure 6:**
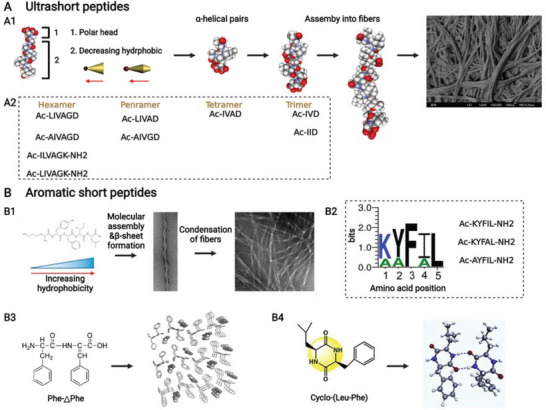
A,B) Ultrashort peptides and aromatic short peptides from amphiphilic peptides. A1) Design principle of ultrashort peptide coupled with its assembly to nanofiber and nanofiber scaffold (scanning electron microscopic image), and A2) some peptide sequences of ultrashort peptides. Reproduced with permission.^[^ ^83^ ^]^ Copyright 2011, Elsevier. B1) Design principle of an aromatic short pentapeptide and its assembly to nanofibers and nanofiber scaffold (transmission electron microscopic image). B2) The residue order of hydrogelating pentapeptides among which the letter size is related to the predominance of residues in pentapeptides gelling under any pH condition, and F and L must be retained for gelation. And hydrogelating peptide sequences of aromatic short pentapeptides in PBS at pH 7.4. Reproduced with permission.^[^ ^86^ ^]^ Copyright 2019, American Chemical Society. B3) Structure of aromatic F—△F dipeptide and its assembly model. Reproduced with permission.^[^ ^89a^ ^]^ Copyright 2007, Wiley‐VCH. B4) Structure of aromatic L—F cyclic dipeptide and its assembly model. Reproduced with permission.^[^ ^90a^ ^]^ Copyright 2019, Elsevier. Created with BioRender.com.

Ultrashort peptides are composed of 3–6 aliphatic amino acids that could be divided into two sections: 1) a relatively hydrophilic amino acid and 2) a long decreasing hydrophobic peptide (**Figure** **6**A1).^[^ ^83^ ^]^ In suitable aqueous solutions, monomers first form *α*‐helical antiparallel pairs, and then peptide pairs assemble to *β*‐turn nanofibers, which further assemble into stable hydrogel (Figure 6A2).^[^ ^83^ ^]^ Among them, hexapeptides tended to form hydrogels more readily than pentapeptides, tetrapeptides, and tripeptides, and stronger hydrogels were obtained by controlling head amino acids from acidic (D and E) to neutral (S and T) and basic aliphatic (K) residues.^[^ ^84^ ^]^ To functionalize ultrashort peptides, their terminals can be modified to allow for the introduction of bioactive motifs by chemical tethering. For instance, an ultrashort peptide (CH_3_CO—LIVAGKC) was fabricated and mixed with CRGD motif for cell adhesion, and CRGD could be conjugated to the ultrashort peptide by disulfide bond after being oxidated overnight by H_2_O_2_.^[^ ^85^ ^]^ So, functional ultrashort peptide‐based hydrogels may be new SPNHs for bone tissue engineering, and they need further investigation.

#### 2.2.4 Aromatic Short Peptides

Aromatic short peptides consisted of 2–5 amino acids from aromatic and aliphatic groups. Tang et al.^[^ ^86^ ^]^ showed a class of pentapeptides that could be assumed to two sections: 1) a peptide that features hydrophilic N‐terminal residues and hydrophilic C‐terminal residues and 2) a relatively hydrophilic head (Figure 6B1,B2). The structure of the hydrophobic section showed increasing hydrophobicity, which is absolutely in contrast with that of ultrashort peptides.^[^ ^86^ ^]^ Phenylalanine at position 3 and leucine at position 5 are highly needed for gelation at any conditions, and tyrosine at position 2 is required for neutral gelation: namely, CH_3_CO—KYFIL—NH_2_, CH_3_CO—AYFIL—NH_2_, and CH_3_CO—KYFAL—NH_2_ could assemble into *β*‐sheet nanofiber hydrogels under neutral conditions.^[^ ^86^ ^]^ These aromatic short peptides show great promise to be used for bone tissue engineering by injectable hydrogels, because it has been revealed that the SPNH assembled by CH_3_CO—AYFIL—NH_2_ supported higher cell viability than phosphate buffered saline (PBS) delivery after injection.^[^ ^86^ ^]^ In recent years, some tripeptides, including KYF^[^ ^87^ ^]^ and DFY,^[^ ^88^ ^]^ were reported to have a possibility to assemble into nanofiber hydrogel, but their relationship with pentapeptides is still unknown. Furthermore, some dipeptides (linear or cyclic) derived from F—F could also assemble into SPNHs. F—△F (△F refers to an *α*,*β*‐dehydrophenylalanine residue, which allows for conformational constraints) is a canonical example which is artificially constructed to improve enzymatic resistant properties, and it could assemble into nanofiber hydrogel without observable cytotoxicity (Figure 6B3).^[^ ^89^ ^]^ Besides, some amino acids, such as ser (S), cys (C), glu (G), his (H), lys (K), and leu (L), could be directly linked to phe (F) to fabricate cyclic dipeptides.^[^ ^90^ ^]^ As an example, in aqueous solutions, cyclic LF assembles into nanofiber hydrogels by hydrogen bonds from cycles and *π*–*π* stacking from benzene rings (Figure 6B4).^[^ ^90a^ ^]^ Although cyclic dipeptides could form SPNHs, whether they can be used as ECM‐like scaffolds is still unknown.

### 2.3 Multirepeat Helical Peptides

In addition to the above short supramolecular peptides, some long supramolecular peptides could be designed on the basis of some natural helical polymers. Considering that they are fabricated by repeated units, they are called multirepeat helical peptides, including *α*‐helical coiled‐coil peptides, *β*‐spiral elastin‐like polypeptides (ELPs), and triple‐helical collagen‐mimetic peptides (CMPs).

#### 2.3.1 *α*‐Helical Coiled‐Coil Peptides

**Figure 7:**
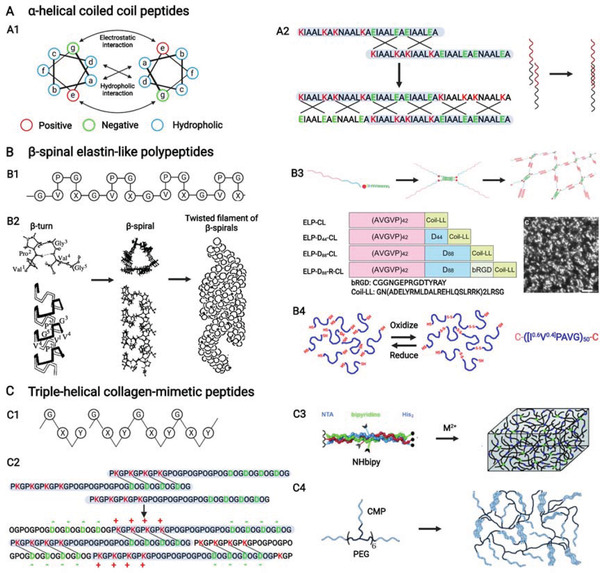
A–C) *α*‐Helical coiled coil peptides, *β*‐spinal elastin‐like polypeptides, and triple‐helical collagen‐mimetic peptides from multirepeat helical peptides. A1) Helical wheel presenting the design principle of coiled‐coil peptides. Reproduced with permission.^[^ ^91^ ^]^ Copyright 2017, Wiley‐VCH. A2) Peptide sequence of hydrogelating *α*‐helical coiled coil peptide with sticky end and its assembly model. B1) Conserved pentapeptide repeat, (VPGXG)*_n_*, of *β*‐spinal elastin‐like polypeptides and B2) structure of (VPGVG)*_n_* coupled with its assembly to nanofibers. Reproduced with permission.^[^ ^95^ ^]^ Copyright 1997, American Chemical Society. B3) Structure and sequence of hybrid elastin‐like peptides combined with assembly into nanofiber scaffold (transmission electron microscopic image). Reproduced with permission.^[^ ^98^ ^]^ Copyright 2020, American Chemical Society. B4) Chemical cross‐links of elastin‐like polypeptides to form rigid hydrogel. Reproduced with permission.^[^ ^99^ ^]^ Copyright 2016, American Chemical Society. C1) Tripeptide repeat, (GXY)*_n_*, of triple‐helical collagen‐mimetic peptides. C2) Peptide sequence of hydrogelating collagen‐mimetic peptide with sticky end and its assembly model. Reproduced under the terms of the CC‐BY license.^[^ ^104^ ^]^ Copyright 2021, The authors, Published by MDPI. C3) Physical cross‐links of collagen‐mimetic peptides by mental ions to form hydrogel. Reproduced with permission.^[^ ^106^ ^]^ Copyright 2009, Wiley‐VCH. C4) Structure of a hybrid collagen‐mimetic peptide and its assembly model. Reproduced under the terms of the CC‐BY license.^[^ ^109^ ^]^ Copyright 2020, The authors, Published by MDPI. Created with BioRender.com.

*α*‐Helix is the most common protein secondary structure in nature, with 3.6 amino acids forming a helix. Thus, a hydrophobic residue needs to be added every on three or four hydrophilic amino acids to promote the formation of *α*‐helix. However, *α*‐helical peptides could not form stable hydrogel because of unstable thermodynamics. Therefore, researchers pay further attention to coiled coils formed by two or more twisted *α*‐helices, characterized by a repeating unit of seven residues denoted as (abcdefg)*_n_* (**Figure** **7**A1).^[^ ^91^ ^]^ The assembling forces derived from points a, d, e, and g are as follows: 1) points a and d are typically strong hydrophobic amino acids but not always, drawing close to each other in an aqueous solution, while 2) points e and g are typically charged amino acids, which may stabilize the assembling structure by electrostatic interactions and promote nanofiber formation by stick ends.^[^ ^92^ ^]^ Points b, c, and f are residues modulating interpeptide interactions and allowing installation of functional groups. In particular, SPNHs could be obtained when points b, c, and f are constituted by weak and extensively cross‐linked amino acids (i.e., alanine and glutamine), and the authors named them as hydrogelating self‐assembling fiber (hSAFs, Figure 7A2).^[^ ^93^ ^]^ Two amino acids were chosen because alanine could promote weak hydrophobic interactions between fibers, while glutamine tends to form hydrogen bonds.^[^ ^93^ ^]^ Contrary to hSAF_QQQ_ peptide1 and hSAF_QQQ_ peptide2, which were constructed by glutamine residues, the hSAF_AAA_ peptide1 (hSAF_AAA_p1) and hSAF_AAA_ peptide2 (hSAF_AAA_p2) that were constructed by alanine residues were suitable for biomedical application because they formed a weak hydrogel at low temperature and a stable hydrogel at room temperature.^[^ ^93^ ^]^ For functionalization, one lysine residue of hSAF_AAA_ peptide1 was substituted by an azido norleucine to fabricate hSAF_AAA_p1(N_3_), which could also assemble with hSAF_AAA_p2 to form a hydrogel; then, an alkyne‐bearing RGDS was appended to the hydrogel by copper‐catalyzed azide–alkyne cycloaddition to endow the SPNH with the function of cell adhesion.^[^ ^94^ ^]^

#### 2.3.2 *β*‐Spiral Elastin‐Like Polypeptides

Elastin is one abundant protein in the human body that is characterized by a conserved pentapeptide repeat, (VPGXG)*_n_*, where X refers to other amino acids except proline (Figure 7B1). Thus, ELPs were mainly fabricated on the basis of (VPGXG)*_n_*, which formed a *β*‐turn structure induced by Pro–Gly, and the repeating *β*‐turns then formed a helical conformation called *β*‐spiral and twisted *β*‐spiral (Figure 7B2).^[^ ^95^ ^]^ The aggregation of ELPs was facilitated when the temperature was above a specific range called inverse phase transition temperature (Tt).^[^ ^96^ ^]^ The Tt below physiological temperature may be useful for bone tissue engineering because this property endow ELPs with injectability. Namely, ELPs can be prepared in solutions below Tt, and then ELPs can assemble into hydrogels when injected to lesions in vivo because the physiological temperature is higher than the Tt of ELPs. The residue X exerted critical effects on the amphiphile of ELPs, which may determine the coacervated structures.^[^ ^97^ ^]^ ELPs could be connected to one another to control aggregation and obtain rigid hydrogels. Two strategies, such as hybrid peptide and cross‐linking strategies, have been developed to link ELPs. Hybrid peptide strategies refer to incorporating ELPs to other hydrogelating biomaterials. Mizuguchi et al.^[^ ^98^ ^]^ designed and synthesized several hybrid peptides by introducing an ELP to a coiled‐coil peptide with or without a negatively charged poly(aspartic acid) for the controlled release of positively charged bioactive factors and a CAP (CGGNGEPRGDTYRAY) derived from bone sialoprotein for cell adhesion through genetic engineering. These peptides could form stable hydrogels because of the assembly of ELP and coiled‐coil peptide (Figure 7B3).^[^ ^98^ ^]^ In terms of cross‐linking strategies, chemical cross‐links are commonly used. For instance, serine was introduced to both terminuses of an ELP, forming robust hydrogels after telechelic oxidative coupling which even stimulates the osteogenic differentiation of MSCs without osteogenic molecules (Figure 7B4).^[^ ^99^ ^]^

At present, almost all ELPs are synthesized by genetic engineering for long peptide sequence, which is not reasonable for solid‐phase synthesis. Therefore, the possibility of constructing short ELPs, which could form sticky ends to assemble into SPNHs, is an interesting topic. A promising hope is to realize this possibility, because ELPs could tolerate relatively broad sequence diversity without losing thermoresponsive properties.^[^ ^100^ ^]^

#### 2.3.3 Triple‐Helical Collagen‐Mimetic Peptides

Collagen is the most abundant protein in the human body, and it is multihierarchically assembled by long peptide strands. It is also characterized by a canonical tripeptide unit, G—X—Y or X—Y—G, where X usually refers to proline (P), and Y refers to hydroxyproline (O) (Figure 7C1).^[^ ^101^ ^]^ Most CMPs are mainly composed of (GPO)*_n_* or (POG)*_n_*, and they fail to assemble into hydrogels. Thus, various gelation strategies have been developed for CMPs to assemble into hydrogels, including sticky‐end, cross‐linking, and hybrid peptide strategies.

Sticky‐end CMPs could be divided into three sections: a positively charged terminus, a POG repeated middle, and a negatively charged terminus. In aqueous solutions, the strong electrostatic interactions and hydrogen bonds may facilitate sticky‐end CMPs to assemble into collagen‐like periodic nanofibers, which may further cross‐link into hydrogels. (PRG)_4_(POG)_4_(EPG)_4_ is the first type of sticky‐end CMP, but it only assembles into periodic fibers that could not form hydrogels.^[^ ^102^ ^]^ One possible reason for this phenomenon is that the side chain of arginine forms a robust hydrogen bond with the backbone carbonyl of an adjacent peptide chain, which constrains it from interacting intimately with glutamate.^[^ ^103^ ^]^ Therefore, a new sticky‐end CMP, (PKG)_4_(POG)_4_(DOG)_4_, was fabricated by lysine–aspartate interaction; it could form salt‐bridged hydrogen bonds, and the peptide could assemble into nanofibers and further aggregate facially and laterally into hydrogels in a manner that simultaneously simulates the natural self‐assembly of collagen at all levels (Figure 7C2).^[^ ^103^, ^104^ ^]^ Considering that collagen is one of the most abound organic components of bone tissue, SPNHs assembled by sticky‐end CMPs show brilliant perspective for bone tissue engineering.

Cross‐linking strategies need additional components to be introduced for CMPs to be chemically or physically cross‐linked. As an example for chemical cross‐linking strategies, cysteine residues could be introduced CMPs because they could form disulfide bond after oxidation.^[^ ^105^ ^]^ In terms of physical cross‐linking strategies, metal ligands that include a nitrilotriacetic acid, a His2 sequence, and a bipyridyl moiety were introduced to the N‐terminus, C‐terminus, and center of a CMP called NHbipy, and the peptide could be triggered by metal ion to assemble into hydrogels (Figure 7C3).^[^ ^106^ ^]^ The middle bipyridyl moiety or the N‐terminus nitrilotriacetic acid could be replaced by bioactive motifs such as RGD (the commonly used CAP) to endow the SPNH with the function of cell adhesion to emulate natural ECM.^[^ ^107^ ^]^

The hybrid peptide strategies for CMPs are conducted by direct synthesis and postchemical modification. For direct synthesis, Luo and Tong^[^ ^108^ ^]^ introduced a CMP [(GPO)_3_GFOGER(GPO)_3_] as a bioactive motif to a PA. The CMP section first assembled into triple‐helical structures, and the supramolecular peptide further assembled into hydrogels on the basis of the hydrogelating mechanisms of PAs.^[^ ^108^ ^]^ For postchemical modification, CMPs are generally linked to PEG to fabricate hybrid CMPs. Balion et al.^[^ ^109^ ^]^ covalently tethered a CMP or a CMP containing RGD to PEG, and a fibril network could be formed by the assembly of CMPs (Figure 7C4).

Although the above three strategies allow CMPs to form hydrogels, combining hybrid CMP strategies and stick‐end strategies is recommended because the assembly of CMPs serves as a cross‐linking to avoid uncontrollable cross‐linking reactions or additional cross‐linkers.^[^ ^110^ ^]^ The mechanical properties and antiprotease stabilities could be also enhanced. Most importantly, bioactive motifs, such as RGD and isoleucine‐lysine‐valine‐alanine‐valine (IKVAV), could be conjugated to CMPs.^[^ ^111^ ^]^

## 3 Biochemical Functionalization for Multifunctional Microenvironment

**Figure 8:**
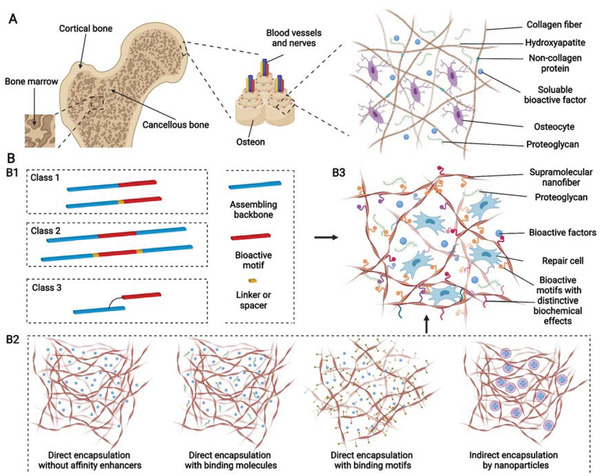
A,B) Schematic of natural hierarchical structures of bone and biochemical functionalization of bioactive supramolecular peptide nanofiber hydrogels to emulate natural bone extracellular matrix. (A) At macroscopic level, bone tissue could be categorized into cancellous bone which is filled with bone marrow and cortical bone which is at the outer surface. At microscopic level, cortical bone is composed of tightly packed osteons, basic units of bone, which are made up of central Harvasian canals containing blood vessels, nerves, and surrounding lamellae characterized by layered collagen fibers. At nanoscopic level, bone cells reside in a highly mineralized collagen matrix containing proteoglycan and bioactive factors. B1) Bioactive supramolecular peptides could be classified into three groups in accordance with the covalently linked site bioactive motifs. B2) Bioactive factors could be directly encapsulated into hydrogel matrix with or without affinity enhancers or indirectly adsorbed with the help of nanoparticles. B3) Multiple functional supramolecular peptide‐tethered distinctive motifs coassemble into nanofiber hydrogels with physical absorption of bioactive factors, which could fulfill the complexity of natural extracellular matrix to the most extent. Created with BioRender.com.

Bones could be categized into cortical and cancellous bones; cortical bone is constructed using tightly packed osteons that are composed of Harvasian canals containing blood vessels and nerve tissue are surrounded by concentric lamella of collagen fibers reinforced by hydroxyapatite (HAP) or noncollagenous structural proteins, including laminin and fibronectin.^[^ ^112^ ^]^ Osteocytes reside in the lacunas of osteons, which also contain proteoglycan and bioactive factors (**Figure** **8**A).^[^ ^113^ ^]^ SPNHs are reminiscent of natural ECM because of their high water content and porous and nanoscale structure. SPNHs are not only promising 3D scaffolds for tissue engineering but also excellent drug‐controlled‐release carriers. Bioactive motifs (bioactive peptides, glycopeptides, or drugs) inducing biochemical signals could be covalently conjugated to fabricate functional supramolecular peptides by initial direct synthesis and postclick chemistry to further simulate the biochemical function of bony ECM. In accordance with the connection site, functional supramolecular peptides could be divided into three groups: connection to the side chain of the assembling backbones without or with a spacer, insertion to the middle of the assembling backbones, and connection to the side chain of amino acids on the assembling backbones (Figure 8B1). However, the incorporation of bioactive motifs may disturb the assembling behavior of supramolecular peptides. Thus, functional supramolecular peptides should coassemble with their basic peptides at certain ratios. Different functional supramolecular peptides containing distinctive motifs could also be incorporated into one SPNH by coassembling. Meanwhile, bioactive factors (bioactive proteins, extracellular vesicles (EVs), or bioactive clinical drugs) could also be physically adsorbed into the hydrogel matrix by direct encapsulation with or without affinity enhancers and indirect encapsulation by nanoparticles (Figure 8B2). And various parameters influence the release of bioactive factors from SPNHs, which mainly include physical hindrances determined by peptide hydrogel density as well as the size of bioactive factors, and noncovalent interactions between the matrix and incorporated bioactive factors.^[^ ^21^, ^114^ ^]^ Therefore, a biomimetic multifunctional microenvironment could be constructed using SPNHs through the above two distinctive strategies (Figure 8B3).

**Figure 9:**
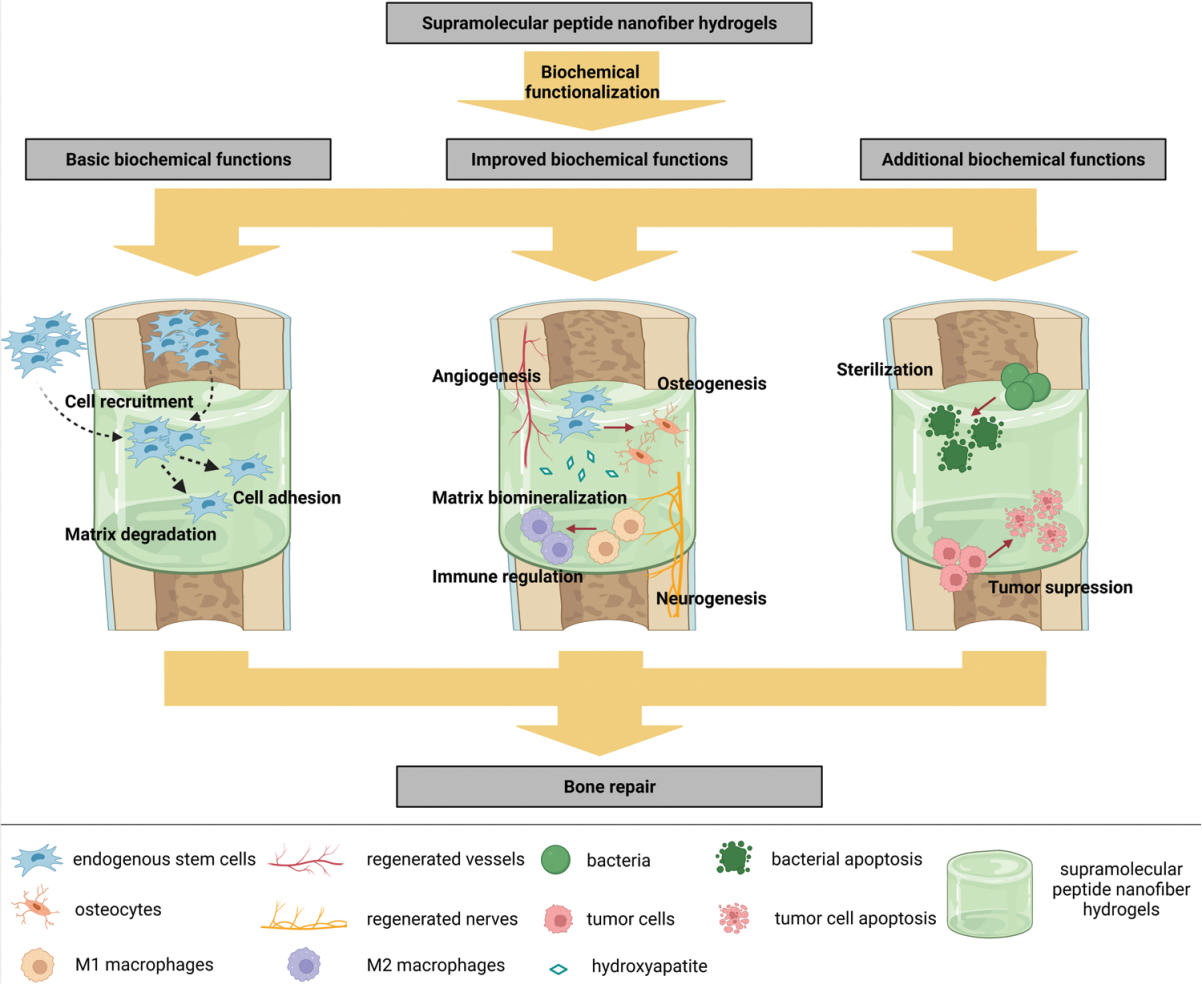
Biochemical functionalization of supramolecular peptide nanofiber hydrogels for multifunctional microenvironment, including basic biochemical functions (cell adhesion, cell recruitment, and matrix degradation), improved biochemical functions (matrix biomineralization, osteogenesis, angiogenesis, neurogenesis, and immune regulation), and additional biochemical functions (sterilization and tumor suppression). Created with BioRender.com.

For successful and satisfying bone healing, sufficient biochemical functions are needed to be introduced SPNHs by conjugating bioactive motifs and absorbing bioactive factors, and these biochemical functions can be categorized into basic biochemical functions (cell adhesion, cell recruitment, and matrix degradation), improved biochemical functions (matrix biomineralization, osteogenesis, angiogenesis, neurogenesis, and immune regulation), and additional biochemical functions (sterilization and tumor suppression) (**Figure** **9**). When SPNHs are implanted into the body, cell adhesion is the most basic function which allows exogenous or endogenous repair cells to survive, proliferate, and differentiate. For emerging cell‐free scaffolds, it is highly needed to recruit endogenous repair cells. And modulating the degradation of SPNHs to collaborate with bone tissue regeneration is also needed during bone repair. These three functions (cell adhesion, cell recruitment, and matrix degradation) apply not only to bone tissue engineering, but also to broader tissue engineering or regenerative medicine, so we term them as basic biochemical functions. For bone tissue regeneration, matrix biomineralization and osteogenesis are needed for the deposition of HAP and collagen. Besides, angiogenesis and neurogenesis are also required because regenerated blood vessels could transport cells, nutrients, and oxygen, and regenerated nerve fibers could promote osteogenesis by secreting neuropeptides. Meanwhile, immune cells also exert indispensable effects in the bone regenerative microenvironment, and especially the proper transition of macrophage phenotype could promote osteogenesis. So, we classify matrix biomineralization, osteogenesis, angiogenesis, neurogenesis, and immune regulation as improved biochemical functions for bone tissue engineering, and a proper balance should be kept among them: matrix biomineralization and osteogenesis are the main processes during bone regeneration which should be moderately supported or enhanced by proper angiogenesis, neurogenesis, and immune regulation. Furthermore, the microenvironment of bone defects caused by the excision of infection or tumor is not suitable for bone regeneration, and it is also needed to avoid postoperative infection and tumor recurrence. So, sterilization and tumor suppression are termed as additional functions from the point of therapeutic purpose. Considering that each bioactive motif or bioactive factor shows multiple effects, they were mainly classified in accordance with their primary function. Various bioactive motifs and bioactive factors are summarized in **Table** **1**.

**Table 1:** Bioactive motifs and factors for bone tissue engineering

Main function	Motifs or factors	Other functions	Usage mode	Ref.
Cell adhesion (bioactive motifs)	RGD, RGDS, PRGDSGYRGDS (PRG), DGRGDSVAYG (DGR), CGGNGEPRGDTYRAY (bRGD)	Osteogenesis, neurogenesis, angiogenesis	Covalent conjugation	^[^ ^82^, ^98^, ^117^, ^119^, ^179^, ^180^ ^]^
	PHSRN	–	Covalent conjugation	^[^ ^118^, ^119^ ^]^
Cell recruitment (bioactive factors)	Stromal cell‐derived factor 1*α*(SDF‐1*α*)	–	Physical absorption	^[^ ^128^ ^]^
	Interleukin‐8 (IL‐8)	–	Physical absorption	^[^ ^129^ ^]^
Cell recruitment (bioactive motifs)	PFSSTKT (bone marrow homing peptide 1, BMHP1), SKPPGTSS (BMHP2)	Osteogenesis, neurogenesis	Covalent conjugation	^[^ ^122^, ^123^, ^222^ ^]^
	RPKPQQFFGLM (substance P, SP)	Osteogenesis, angiogenesis	Physical absorption, covalent conjugation	^[^ ^125^, ^223^ ^]^
	KPSSAPTQLN (KPSS)	Anti‐inflammatory and antiapoptotic effects	Covalent conjugation	^[^ ^126^, ^224^ ^]^
	EPLQLKM (E7)	Osteogenesis	Covalent conjugation	^[^ ^127a–c^ ^]^
Matrix degradation (bioactive motifs)	GPQGIWGQ	Motif delivery	Covalent conjugation	^[^ ^134^ ^]^
	GTAGLIGQ, GPQGIASQ, GPQGPAGQ, GPLGIAQG, PVGLIG, LRG	Motif delivery	Covalent conjugation	^[^ ^131^, ^132^, ^225^ ^]^
	PLGL	Motif delivery	Covalent conjugation	^[^ ^135^ ^]^
	PTGXKV	Motif delivery	Covalent conjugation	^[^ ^133^ ^]^
	LIKMKP	Motif delivery	Covalent conjugation	^[^ ^226^ ^]^
Matrix biomineralization (bioactive motifs)	^p^S, ^p^SD^p^S	Osteogenesis	Covalent conjugation	^[^ ^72^, ^136^ ^]^
	EEE, EEEEE	Osteogenesis	Covalent conjugation	^[^ ^138^, ^140^, ^171^ ^]^
	DDDDD	–	Covalent conjugation	^[^ ^138c^ ^]^
	NPYHPTIPQSVH	HAP binding	Covalent conjugation	^[^ ^79^ ^]^
	MLPHHGA	HAP binding	Covalent conjugation	^[^ ^142a^ ^]^
	SVSVGMKPSPRP	HAP binding	Covalent conjugation	^[^ ^143a^ ^]^
	STLPIPHEFSRE	HAP binding	Covalent conjugation	^[^ ^144^ ^]^
	VTKHLNQISQSY, VTKHLNQISPQSPY, VTKHLNEISQSY, IVQSKHTLSNQY	HAP binding	Covalent conjugation	^[^ ^144^, ^145^ ^]^
Osteogenesis (bioactive factors)	Bone morphogenetic proteins (BMPs)	Angiogenesis	Physical absorption	^[^ ^2^, ^227^ ^]^
	Lactoferrin	–	Physical absorption	^[^ ^149^ ^]^
	Parathyroid hormone (PTH)	Angiogenesis	Physical absorption	^[^ ^150^ ^]^
	Parathyroid hormone‐related protein (PTHrP)	Angiogenesis	Physical absorption	^[^ ^151^ ^]^
	Osteoinductive drugs, such as aspirin, simvastatin, bisphosphonate, etc.	–	Physical absorption	^[^ ^187^, ^188^, ^203^ ^]^
	Extracellular vesicles (EVs)	Angiogenesis	Physical absorption	^[^ ^184^, ^228^ ^]^
Osteogenesis (bioactive motifs)	LRKKLGKA	To indirectly interact with BMP‐2 by heparan sulfate to enhance the osteogenesis	Covalent conjugation	^[^ ^152^, ^153^, ^154^ ^]^
	3,4,6S‐GlcNAc	To directly interact with BMP‐2 to enhance osteogenesis	Covalent conjugation	^[^ ^155^ ^]^
	TSPHVPYGGGS	To directly interact with BMP‐2 to enhance osteogenesis	Covalent conjugation	^[^ ^157^ ^]^
	KIPKASSVPTELSAISTLYL (P20), pSKIPKASSVPTELSAISTLYLDDD (P24), SpSVPTNSPVNSKIPKACCVPTELSAI (BMP‐2‐mimetic peptide)	–	Covalent conjugation/physical absorption	^[^ ^159^, ^160^, ^161^, ^229^ ^]^
	RKKNPNCRRH (BMP‐4‐mimetic peptide)	–	Covalent conjugation/physical absorption	^[^ ^162^ ^]^
	GQGFSYPYKAVFSTQ (BMP‐7‐mimetic peptide)	–	Covalent conjugation/physical absorption	^[^ ^163^, ^230^ ^]^
	CGGKVGKACCVPTKLSPISVLYK (BMP‐9‐mimetic peptide)	–	Covalent conjugation/physical absorption	^[^ ^164^ ^]^
	SVSEIQLMHNLGKHLNSMERVEWLRKKLQDVHNF (Teriparatide), pSVSEIQLMHNLGKHLNSMERVEWLRKKLQDVHNFDDD (PTHrP1), pSVSEIQLMHNLGKHLNSMERVEWLRKKLQDVHNFEEE (PTHrP2)	Angiogenesis	Covalent conjugation/physical absorption	^[^ ^166^ ^]^
	AVSEHQLLHGKGKSIQDLRRRFFLHHLIAEIHTAEIR (PTHrP1‐37), AVSEHQLLHGKGKSIQDLRRRELLEKLLXKLHTA (Abaloparatide)	Angiogenesis	Covalent conjugation/physical absorption	^[^ ^167^, ^168^ ^]^
	TRSAW (PTHrP107‐111)	–	Covalent conjugation	^[^ ^169^ ^]^
	RKVRGPPVSCIKRDSPIQ (LP2)	–	Covalent conjugation/physical absorption	^[^ ^170^ ^]^
	DGEA	Selective adhesion for osteoblasts by integrin *α*2*β*1	Covalent conjugation	^[^ ^171^, ^231^ ^]^
	GFOGER	Selective adhesion for osteoblasts by integrin *α*2*β*1	Covalent conjugation	^[^ ^172^ ^]^
	GTPGPQGIAGQRGVV	Selective adhesion for osteoblasts by integrin *α*2*β*1	Covalent conjugation	^[^ ^173^ ^]^
	KRSR	Selective adhesion for osteoblasts by cell‐membrane heparin sulfate proteoglycans	Covalent conjugation	^[^ ^231^ ^]^
	ALKRQGRTLYGFGG (osteogenic growth peptide, OGP), YGFGG (OPG10‐14)	–	Covalent conjugation/physical absorption	^[^ ^179^, ^180^, ^232^ ^]^
	ACDTATCVTHRLAGLLSRSGGVVKNNFVPTNVGS (calcitonin‐gene related peptide, CGRP)	Angiogenesis, neurogenesis	Covalent conjugation/physical absorption	^[^ ^182^ ^]^
Angiogenesis (bioactive factors)	Vascular endothelial growth factor (VEGF)	Osteogenesis	–	^[^ ^190^ ^]^
	Basic fibroblast growth factor (bFGF)	Osteogenesis	–	^[^ ^191^ ^]^
	Insulin‐like growth factors (IGFs)	Osteogenesis	–	^[^ ^192^ ^]^
Angiogenesis (bioactive motifs)	KLTWQELYQLKYKGI (QK or KLT)	–	Covalent conjugation	^[^ ^194^, ^195^ ^]^
	GYGSSSRRAPQT (IGF‐1‐mimetic peptide)	–	Covalent conjugation	^[^ ^196^ ^]^
	EGDK(pbs)S (heparin‐mimetic component)	–	Covalent conjugation	^[^ ^197^ ^]^
	REDV	Selective adhesion for vascular endothelial cells by integrin *α*4*β*1	Covalent conjugation	^[^ ^198^ ^]^
	SVVYGLR	Selective adhesion for vascular endothelial cells by integrin *α*4*β*1 and *α*9*β*1	Covalent conjugation	^[^ ^199^, ^200^ ^]^
Neurogenesis (bioactive factors)	MicroRNA‐222	–	–	^[^ ^203^ ^]^
	Nerve growth factor (NGF)	Osteogenesis	Physical absorption	^[^ ^202^ ^]^
	Brain‐derived neurotrophic factor (BDNF)	Osteogenesis	Physical absorption	^[^ ^204^ ^]^
Neurogenesis (bioactive motifs)	RGIDKRHWNSQ (BDNF‐mimetic peptide)	–	Covalent conjugation	^[^ ^206a^ ^]^
	Cyclic RKKADP (BDNF‐mimetic peptide)	–	Covalent conjugation	^[^ ^206b^ ^]^
	IKVAV, CQAASIKVAV	Cell adhesion by laminin‐binding protein, angiogenesis	Covalent conjugation	^[^ ^205^, ^209^, ^233^ ^]^
	YIGSR, CDPGYIGSR	Cell adhesion by laminin‐binding protein, angiogenesis	Covalent conjugation	^[^ ^117^, ^198^, ^205^ ^]^
	RNIAEIIKDI	Cell adhesion by laminin‐binding protein	Covalent conjugation	^[^ ^234^ ^]^
	EVYVVAENQQGKSKA (FGL)	–	Covalent conjugation	^[^ ^207^, ^235^ ^]^
	SIDRVEPYSSTAQ (FRM)	–	Covalent conjugation	^[^ ^208^ ^]^
	KLPGWSG (KLP)	–	Covalent conjugation	^[^ ^56^ ^]^
	FAQRVPP (FAQ)	–	Covalent conjugation	^[^ ^211^, ^236^ ^]^
	NAVSIQ	–	Covalent conjugation	^[^ ^135^, ^237^ ^]^
Immune regulation	IL‐4	–	–	^[^ ^214^ ^]^
	Rosiglitazone (RSG)	‐–	–	^[^ ^215^ ^]^
Sterilization	Antimicrobial peptides (AMPs)	–	Covalent conjugation/physical absorption	^[^ ^217^ ^]^
	Ciprofloxacin	–	Physical absorption	^[^ ^218^ ^]^
	RRR	–	Covalent conjugation	^[^ ^219^ ^]^
Tumor suppression	Mg particles	Osteogenesis	Physical absorption	^[^ ^220^ ^]^
	Metformin	–	Physical absorption	^[^ ^221^ ^]^

### 3.1 Basic Biochemical Functions

#### 3.1.1 Cell Adhesion

Cell adhesion is the most fundamental function for biomaterials. It could be medicated by CAPs, which could bind to related receptors, such as integrin, laminin binding proteins, and transmembrane proteoglycans, on the cell membrane. Several CAPs target integrin, among which fibronectin‐derived RGD has been widely utilized.^[^ ^115^ ^]^ RGD could bind to multiple integrins and thus further activate other functions, such as osteogenesis, angiogenesis, and neurogenesis.^[^ ^116^ ^]^ The peptide and its derivatives [such as RGDS, PRGDSGYRGDS (PRG), and DGRGDSVAYG (DGR)] have been covalently tailored to various supramolecular peptides to form functional nanofiber hydrogels.^[^ ^117^ ^]^ Proline‐histidine‐serine‐arginine‐asparagine (PHSRN), another fibronectin‐derived CAP, was recently introduced to supramolecular peptides containing RGDS to enhance cell adhesion because of its synergistic effect with RGDS when separated by 3.2 nm but no more than 5.5 nm.^[^ ^118^ ^]^ VECs encapsuled in hydrogels with controlled RGDS—PHSRN spacing of 3.2 nm appeared to be more spread, with an upregulated expression of *α*_5_ *β*_1_ integrin, than hydrogels without the synergetic effect.^[^ ^118a^ ^]^ RGDS and PHSRN could be introduced into hydrogels by coassembling of two peptides carrying RGDS and PHSRN, respectively, thereby extensively enhancing cell adhesion, spreading, and proliferation.^[^ ^119^ ^]^ Therefore, it is recommended to synthetically introduce both RGDS and PHSRN into SPNHs for bone tissue engineering.

#### 3.1.2 Cell Recruitment

Cell recruitment is a process wherein endogenous repair cells from a central cell niche (such as bone marrow) or surrounding tissue are induced to migrate into biomaterials, thus avoiding the encapsulation of exogenous seed cells. SPNHs provide porous ECM‐like microenvironment that supports cell recruitment, and the ability could be promoted by some bioactive motifs. Bone marrow homing peptides (BMHPs), a series of polypeptides rich in K, P, F, S, and T, are identified by phage display screening; they could promote MSC recruitment or migration, among which BMHP1 (PFSSTKT) and BMHP2 (SKPPGTSS) have been widely used.^[^ ^120^ ^]^ Lu et al.^[^ ^121^ ^]^ fabricated composite nanoscaffolds by combining decellularized cartilage matrix (DCM) and RADA16 hydrogel or RADA16/RADA16—GG—PFSSTKT hydrogel, and the homing results in vivo showed that the composite nanoscaffold containing RADA16/RADA16—GG—PFSSTKT hydrogel show more CD29+/CD90+ dual positive MSCs than other groups. Similar recruitment results were also reported in RADA16/RADA16—GG—SKPPGTSS hydrogel.^[^ ^122^ ^]^ And both RADA16/RADA16—GG—PFSSTKT hydrogel and RADA16/RADA16—GG—SKPPGTSS hydrogel could enhance subchondral bone regeneration when they are integrated into DCM.^[^ ^121^, ^122^ ^]^ Therefore, we believe that promising bone repair results may be obtained if they are loaded to other carriers such as decellularized bone matrix. Furthermore, it has been revealed that BMHP1, when tethered to RADA16, could stimulate the osteogenic differentiation of MSCs with higher ALP activity and improved osteogenic gene expression, and the RADA16/RADA16—GG—PFSSTKT hydrogel could promote the healing of rat cranial defects.^[^ ^123^ ^]^ The possible reason to interpretate the osteoinductivity of RADA16—GG—PFSSTKT may be that lysine residue in BMHP1 and arginine residues in RADA16 synthetically induce stronger electrostatic interaction with BMP receptor 1A than BMP‐2.^[^ ^123^ ^]^ But whether BMHP2 shows osteoinductive activity when tethered to RADA16 remains unknown. Besides, substance P (SP, RPKPQQFFGLM) is a neuropeptide that could recruit MSCs.^[^ ^124^ ^]^ Kim et al.^[^ ^125^ ^]^ subcutaneously implanted PLA scaffolds combined with PBS, KLD12 hydrogel, or KLD12/KLD12—SP hydrogel to nude mice, and found that KLD12‐/KLD12—SP‐hydrogel‐contained polylactic acid (PLA) scaffold shows the best homing activity because it recruits the most labeled MSCs, and KLD12‐/KLD12—SP‐hydrogel‐contained scaffold was reported to promote bone regeneration and bone integration in vivo. Other peptides that could recruit stem cells, including KPSS (KPSSAPTQLN)^[^ ^126^ ^]^ and E7 (EPLQLKM),^[^ ^127^ ^]^ also showed promising prospect to be tailed to supramolecular peptides for bone tissue engineering, and they need further investigation. Besides, some bioactive proteins (such as stromal cell‐derived factor 1*α* (SDF‐1*α*)^[^ ^128^ ^]^ and interleukin‐8 (IL‐8)^[^ ^129^ ^]^) showed cell recruitment property, thus shedding light on the physical encapsulation into SPNHs for bone repair.

#### 3.1.3 Matrix Degradation

Compatible with cell recruitment, the hydrogel matrix should show cell‐mediated degradable sites for endogenous repair cells to infiltrate. Meanwhile, the rate of matrix degradation should maintain balance with the speed of tissue regeneration. When cells migrate into the hydrogels, some enzymes, such as MMPs, may be secreted, thus exerting indistinctive effects on bone regeneration.^[^ ^130^ ^]^ Therefore, the incorporation of MMP‐clearable sequences is an ideal strategy to control hydrogel matrix degradation. GTAGLIGQ was the first type MMP‐2 clearable motif to fabricate cell‐responsive PAs, and type IV collagenase was used to investigate the susceptibility of the SPNH to protease degradation which was assembled by CH_3_(CH_2_)_14_CONH—GTAGLIGQ—ERGDS.^[^ ^77^ ^]^ The results revealed that the SPNH lost 50% weight when incubated with the enzyme in 1 week and was completely degraded within 1 month.^[^ ^77^ ^]^ Other MMP‐2 clearable motifs, such as proline‐valine‐glycine‐leucine‐isoleucine‐glycine (PVGLIG)^[^ ^131^ ^]^ and leucine‐arginine‐glycine (LRG),^[^ ^132^ ^]^ could be inserted to the middle of assembling backbone for matrix degradation. Two strategies could be utilized to control the rate of matrix degradation. One strategy is the application of different motifs which show distinctive reaction rates for MMPs and may cause distinctive mechanical properties of SPNHs. Giano et al.^[^ ^133^ ^]^ introduced different MMP‐13 clearable motifs following the pattern of PTGXKV to *β*‐hairpin peptides where the X position was displaced by different residues: phenylalanine for DP1, leucine for DP2, isoleucine for DP3, and alanine for DP4. The results revealed that different *β*‐hairpin peptides showed distinctive degradation rates following DP1 > DP2 > DP3 > DP4, which was slightly different from the hypothesis of DP2 > DP3 ≈ DP1 > DP4.^[^ ^133^ ^]^ Possible explanation for this discrepancy may be the least mechanical property of DP1 which may allow MMP‐13 to penetrate and degrade the matrix more easily.^[^ ^133^ ^]^ So, the results of cellular invasion assay revealed that more migrated cells were detected in DP3 than DP1.^[^ ^133^ ^]^ The other strategy is to link MMP clearable motifs by different spacers with distinctive secondary structures. An MPP‐1 clearable motif (GPQGIWGQ) was linked to a hydrophobic alkyl chain by different spacers with distinctive secondary structures: PA1, PA3, PA4 (well‐known *β*‐sheet sequences), and PA2 (sequence revealing 20% helix).^[^ ^134^ ^]^ The results showed that no degradation was observed for PA3 and PA4 nanofibers within 72 h, and bare degradation was detected for PA1 nanofibers in 24 h (0.21%).^[^ ^134^ ^]^ However, the degradation was obviously enhanced for PA2, namely, 3.22% degradation was displayed after 24 h.^[^ ^134^ ^]^ These results suggested that the secondary structures of spacer could interfere with the degradability by affecting the active‐site availability.^[^ ^134^ ^]^

Besides, the enzyme clearable motifs could be used to deliver other biochemical motifs, which may allow for the controlled release in response to cell behavior. For example, an MMP‐9 clearable motif (PLGL) was incorporated between a sulfo‐group‐functionalized assembling backbone and a neuroprotective hexapeptide.^[^ ^135^ ^]^ At the site of injury, the PLGL motif could be cleared by overexpressed MMP‐9 for the neurotrophic factor to be released to the hydrogel matrix.^[^ ^135^ ^]^ Both results in vitro and in vivo showed that the functional hydrogel is biocompatible, which could stimulate neurite outgrowth and increase the expression of critical neurogenic markers.^[^ ^135^ ^]^

### 3.2 Improved Biochemical Functions

#### 3.2.1 Matrix Biomineralization

Matrix biomineralization refers to the deposition of HAP in biomaterial substrates, and it is beneficial for osteogenesis. Acid amino acids are generally used for matrix biomineralization. Phosphoserine (^P^S) enriched in skeletal saliva protein and dentin phosphoprotein is currently the most widely used biomineralization motif, and it could attract calcium ions and induce nucleation by ALP‐released phosphoric acids.^[^ ^136^ ^]^ PA nanofibers assembled by CH_3_(CH_2_)_14_CONH—CCCCGGG—^P^SRGD were subjected to the mixed solution of 10 × 10^−3^ m CaCl_2_ and 5 × 10^−3^ m Na_2_HPO_4_, and mineral crystals were then obtained on the 2D surface of nanofibers after 30 min, and the Ca/P ratio tested by energy dispersion X‐ray fluorescence spectroscopy was 1.67 ± 0.08 which is in accord with HAP, namely, Ca_10_(PO_4_)_6_(OH)_2_.^[^ ^72^ ^]^ And it was also found that the crystallographic *c*‐axis of mineralized HAP was parallel with the long axis of PA nanofibers, which is reminiscent of the natural alignment of HAP to collagen fibers in bone tissue.^[^ ^72^ ^]^ Furthermore, another PA containing ^P^S was induced by culture medium containing Ca^2+^ to form SPNH, which was then incubated by osteoblastic culture medium containing enzyme ALP and supplemented *β*‐glycerophosphate as phosphate provider.^[^ ^137^ ^]^ The results showed that apparent biomineralization was induced throughout the 3D hydrogel matrix after 8 days, and that the mineralized crystals show analogous Ca/P ratio (1.675 ± 0.015), size (50–75 nm wide, 100–200 nm long, and 4–6 nm thick), crystallographic alignment to natural bone mineral.^[^ ^137^ ^]^ Similar to ^P^S, consecutive negative amino acids (such as EEE, EEEEEE, and DDDDD) could direct mineralization when conjugated to different SPNHs.^[^ ^138^ ^]^ For bone tissue engineering, Sargeant et al.^[^ ^139^ ^]^ used SPNHs containing ^P^S or S to 3D culture MSCs by osteogenic induction medium suppled with 20 × 10^−3^ m CaCl_2_, and found that higher ALP gene and lower osteopontin gene were expressed in SPNHs containing ^P^S than those containing S, which suggest that SPNHs containing ^P^S could be directly used as osteoinductive scaffolds without premineralization for bone tissue engineering. Although premineralized SPNHs may stimulate osteogenesis,^[^ ^140^ ^]^ they may not allow for subsequent cell encapsulation or bioactive factor loading by facile mixing. So, it is still highly recommended to directly use SPNHs containing negative biomineralization motifs as biomaterial scaffolds for bone tissue engineering.

In addition to negative biomineralization motifs, some bioactive motifs identified by phage display screening are capable of biomineralization with high affinity to HAP. These motifs may not only integrate the organic–inorganic matrix but also template the growth of HAP. For example, a CMP (NPYHPTIPQSVH) showed a sequence similar to the basic unit of type I collagen (Gly–Pro–Hyp), which could bind to HAP and direct the nucleation and growth of HAP.^[^ ^141^ ^]^ The peptide was then tethered to a long fatty acid to fabricate a functional PA named collagen‐like PA (CLPA), and it was found that the self‐templated CLPA could support the oriented growth of MC3T3‐E1 preosteoblasts and the deposition of HAP on the surface or in the matrix after being treated with mineralized‐induced fluid, a supersaturated mixture of Ca^2+^ and PO_4_^3−^ with or without poly(aspartic acid) for matrix mineralization.^[^ ^79^ ^]^ Likewise, another peptide called hydroxyapatite binding peptide 1 (HABP1), MLPHHGA, was introduced to MAX8, and the coassembling MAX8—HABP1/MAX8 hydrogel was capable of templating mineralization.^[^ ^142^ ^]^ Other HABPs also showed promise to be covalently bound to supramolecular peptides for bone tissue engineering, including SVSVGMKPSPRP,^[^ ^143^ ^]^ STLPIPHEFSRE,^[^ ^144^ ^]^ and VTKHLNQISQSY and its deviants.^[^ ^144^, ^145^ ^]^ All of these biomineralization motifs identified by phage display screening show great potential to introduced to SPNHs for bone tissue engineering like negative biomineralization motifs which have been thoroughly studied.

#### 3.2.2 Osteogenesis

In addition to mineralization‐derived osteogenic effects, osteogenesis could be modulated by osteoinductive factors or motifs. Recombinant BMPs are the most widely used bioactive proteins, among which at least seven members (BMP‐2, ‐3, ‐4, ‐6, ‐7, ‐9, and ‐12) show osteoinductive properties, and BMP‐2 and BMP‐7 have been used for clinic practice.^[^ ^146^ ^]^ Hosseinkhani et al.^[^ ^147^ ^]^ blended PA stock solution with BMP‐2 suspension to form a stable hydrogel that could release BMP‐2 for up to 25 days in vitro. And the SPNH containing BMP‐2 could effectively induce homogeneous ectopic ossification (a process of homogeneous bone regeneration at soft tissues but not bone) after being injected into the back subcutis of rats.^[^ ^147^ ^]^ Another group implanted RADA16 hydrogel encapsulated with BMP‐2 to rabbit calvaria bone defects, and found that the BMP‐2‐embedded SPNH promoted more newly formed bone tissue than other groups including unfilled group, BMP‐2 solution group, and RADA16 hydrogel group.^[^ ^148^ ^]^ The above studies show that SPNHs may be ideal carriers to deliver osteoinductive factors for bone regeneration. Other osteoinductive proteins have been tested for bone tissue engineering, including lactoferrin,^[^ ^149^ ^]^ parathyroid hormone (PTH),^[^ ^150^ ^]^ and PTH‐related protein (PTHrP).^[^ ^151^ ^]^ While the direct encapsulation of recombinant bioactive proteins could apparently promote bone healing, a plethora of challenging issues exists, such as difficult purification, high price, supraphysiological dose, fast release, susceptible degradation, and random folding. Therefore, multiple strategies have been developed to overcome these issues.

One successful strategy is to improve the affinity between the SPNH matrix and bioactive factors. In natural ECM, heparan sulfate (HS) is one highly sulfated glycosaminoglycans that could noncovalently bind several bioactive proteins by electrostatic interaction and conformational configuration.^[^ ^152^ ^]^ The interactions exert critical effects on potentiating signaling, stabilizing receptors, and avoiding proteolysis.^[^ ^153^ ^]^ In this context, Lee et al.^[^ ^154^ ^]^ fabricated a functional PA containing a heparin binding peptide (LRKKLGKA) to mimic the natural interaction, and then added the peptide stock solutions and the solutions containing BMP‐2 with or without HS into collagen scaffolds to form hydrogels. It was revealed that the addition of HS could remarkably prolong the release of BMP‐2: for initial 24 h, 45.4 ± 8.8% of BMP‐2 was released from SPNH without HS, while only 22.7 ± 3.9% of BMP‐2 was released form SPNH containing HS; for subsquent 8 days, 84.0 ± 18.9% of BMP‐2 was released from SPNH without HS, while 34.5 ± 8.1% was released from SPNH containing HS.^[^ ^154^ ^]^ And it was also found that collagen/hydrogel/BMP‐2/HS composites could effectively decrease the required therapeutic dosage of BMP‐2 by 10 times and promote more bone regeneration than collagen/BMP‐2 implants.^[^ ^154^ ^]^ So, this strategy may effectively reduce therapeutic dosage of BMP‐2 when it is used in the clinic, and related complications caused by high dosages may be further avoided. However, animal‐derived HS has limited clinic application because of poor availability, high heterogeneity, and potential side effects. Thus, sulfated monosaccharides were directly used to mimic natural polysaccharides, which were appended to PAs.^[^ ^155^ ^]^ In a rat posterolateral lumbar intertransverse spinal fusion model, collagen/3,4,6S‐GlcNAc hydrogel/BMP‐2 implants could accomplish comparable regenerated bone volume with collagen/BMP‐2 composites, but the dosage was reduced by 100‐fold.^[^ ^155^ ^]^ Therefore, the trisulfated monosaccharide is capable of mimicking the function of HS, and it could be incorporated with other supramolecular peptides for more investigations.^[^ ^155^ ^]^ Furthermore, some bioactive motifs identified by phage display screening could noncovalently bind bioactive proteins to improve the capacities of controlled release and recruit endogenous bioactive factors.^[^ ^155^, ^156^ ^]^ For instance, TSPHVPYGGGS showed high BMP‐2 binding affinity when conjugated to a PA, and the functional PA was coassembled with basic PA to form a bioactive SPNH.^[^ ^157^ ^]^ Compared with collagen/BMP‐2 implants, the hydrogel containing BMP‐2 binding motif and BMP‐2 could reduce the treatment dose by nearly 10 times, with comparable bone regeneration.^[^ ^157^ ^]^ The hydrogel containing BMP‐2 binding motif without BMP‐2 could induce 42% fusion by recruited bioactive proteins, showing that the motif could recruit endogenous BMP‐2.^[^ ^157^ ^]^ HSNGLPL is another bioactive motif with transforming growth factor‐*β*1 (TGF‐*β*1) binding affinity, and coassembling SPNH containing this motif and TGF‐*β*1 has been used for cartilage regeneration.^[^ ^158^ ^]^ Therefore, more bioactive binding motifs must be displayed for specific proteins.

Another thriving strategy is to tether bioactive‐protein‐related peptides as bioactive motifs to the backbone of supramolecular peptides. A phosphorylated BMP‐2‐related peptide (S^p^SVPT) was incorporated to a PA and then induced to assemble into hydrogel for cell culture and bone repair; the results showed that the functional hydrogel effectively regulated the proliferation and osteogenic differentiation of MSCs in vitro and induced bone regeneration when encapsuled with MSCs.^[^ ^159^ ^]^ P24 (^p^SKIPKASSVPTELSAISTLYLDDD), a peptide developed from a BMP‐2‐related peptide (KIPKASSVPTELSAISTLYL), was tailored to RADA16 for bone tissue engineering.^[^ ^160^ ^]^ Other promising BMP‐related peptides include NSPVNSKIPKACCVPTELSAI from BMP‐2,^[^ ^161^ ^]^ RKKNPNCRRH from BMP‐4,^[^ ^162^ ^]^ GQGFSYPYKAVFSTQ from BMP‐7,^[^ ^163^ ^]^ and CGGKVGKACCVPTKLSPISVLYK from BMP‐9.^[^ ^164^ ^]^ PTH1–34, also called teriparatide (SVSEIQLMHNLGKHLNSMERVEWLRKKLQDVHNF), is the first 34 amino acids at the N‐terminal of PTH. PTH1–34 has been tethered to fibrin hydrogel matrix by a transglutaminase substrate and a plasmin clearable sequence, and the matrix derivatized with PTH1–34 has been confirmed to stimulate bone regeneration.^[^ ^165^ ^]^ Thus, PTH1–34 is a potential bioactive motif to be tailored to supramolecular peptides for bone tissue engineering. Other well‐characterized osteogenic peptides include PTHrP1 (^p^SVSEIQLMHNLGKHLNSMERVEWLRKKLQDVHNFDDD) and PTHrP2 (^p^SVSEIQLMHNLGKHLNSMERVEWLRKKLQDVHNFEEE) from PTH,^[^ ^166^ ^]^ PTHrP1–37 (AVSEHQLLHGKGKSIQDLRRRFFLHHLIAEIHTAEIR),^[^ ^167^ ^]^ abaloparatide (AVSEHQLLHGKGKSIQDLRRRELLEKLLXKLHTA, where X refers to 2‐methylalanine),^[^ ^168^ ^]^ PTHrP107–111 (TRSAW) from PTHrP,^[^ ^169^ ^]^ and lp2 (RKVRGPPVSCIKRDSPIQ) from lactoferrin.^[^ ^170^ ^]^ SPNHs tethered with these bioactive motifs also have potential utility for the treatment of clinic bone defects.

ECM‐derived peptides could also be used as bioactive motifs for osteogenesis. In addition to RGD and its derivates, DGEA,^[^ ^171^ ^]^ GFOGER,^[^ ^172^ ^]^ and P‐15 (GTPGPQGIAGQRGVV),^[^ ^173^ ^]^ which are all derived from type I collagen, could selectively bind osteoblasts by integrin *α*_2_ *β*_1_. Another ECM‐derived peptide that targets osteoblasts is lysine‐arginine‐serine‐arginine (KRSR), an example of heparin‐binding peptide that could bind to HS on osteoblast cell membrane.^[^ ^161^, ^174^ ^]^ The motif was found in five ECM components, including bone sialoprotein, osteopontin, fibronectin, thrombospondin, and vitronectin, and it showed comparable adhesion for osteoblasts with RGD.^[^ ^175^ ^]^ Phenylalanine‐histidine‐arginine‐arginine‐isoleucine‐lysine‐alagine (FHRRIKA) is also a heparin‐binding peptide derived from bone sialoprotein; it could promote biomineralization and improve osteoblast activities.^[^ ^176^ ^]^ Therefore, these ECM‐derived peptides can be used as bioactive alternatives to be conjugated to supramolecular peptides for bone tissue engineering.

Besides, some naturally existing peptides show osteoinductive properties. One widely used motif is osteogenic growth peptide (OGP, ALKRQGRTLYGFGG), which is a native peptide from human serum, regulating the proliferation, differentiation, ALP activity, and matrix mineralization of osteogenic lineage cells.^[^ ^177^ ^]^ And the bioactive sequence of OGP is its C‐terminus, namely, OGP(10–14) (YGFGG).^[^ ^178^ ^]^ Tsutsumi et al.^[^ ^179^ ^]^ used a SPNH assemble by 80% E1Y9 and 20% E1Y9—GGG—ALKRQGRTLYGFGG to 3D culture MC3T3‐E1 preosteoblasts under osteogenic induction medium, and found that higher ALP activity and higher expression of RUNX2 and osteopontin were observed in the SPNH than E1Y9 hydrogel. And higher ALP activity and higher osteocalcin expression were also reported in RADA16/RADA16—GG—ALKRQGRTLYGFGG hydrogel.^[^ ^180^ ^]^ Therefore, SPNHs conjugated with OGP or shorter OPG(10–14) may be attractive scaffolds for bone tissue engineering, and further evidence in vivo is highly needed. Neuropeptides are also naturally existing peptides delivered by nerve fibers from sympathetic/parasympathetic ganglia by axonal transport, and contribute to bone remodeling.^[^ ^181^ ^]^ Among various neuropeptides, calcitonin‐gene‐related peptide and SP promote osteogenesis.^[^ ^182^ ^]^ But few studies pay attention to tethering neuropeptides to SPNHs for bone regeneration.

In addition to osteoinductive signals based on proteins or peptides, EVs were recently recognized as potential alternatives because of their abundant bioactive components (microRNAs, mRNAs, growth factors, chemokines, and cytokines) that mediate intercellular interaction and promote osteogenesis and angiogenesis.^[^ ^183^ ^]^ The receptor activator of nuclear factor kappa‐B and receptor activator of nuclear factor kappa‐B ligand may also be found in some EVs, showing great potential for bone remodeling.^[^ ^184^ ^]^ However, the short half‐life and rapid clearance limit their application for bone tissue engineering. Therefore, excellent biomaterials may be the critical threshold for translational medicine. In accordance with size and biogenesis, EVs could be mainly divided into three classes: exosomes (40–100 nm), microvesicles (100–1000 nm), and apoptotic bodies (50–5000 nm).^[^ ^185^ ^]^ The size of EVs is apparently larger than the pores of SPNHs, and this difference may fulfill the delayed release and protect their bioactivity. Meanwhile, membrane proteins, such as integrins, may exist on the surface of EVs. Thus, functional peptide hydrogel with CAPs may enhance the affinity between the hydrogel matrix and EVs. Therefore, SPNHs may be excellent biomaterials to encapsule EVs for bone regeneration, but related studies have not been reported. EVs loaded in SPNHs were recently used for angiogenesis, anti‐inflammatory, and antifibrosis, showing promising potential for bone repair in the future.^[^ ^186^ ^]^

Considering that SPNHs are an excellent drug deliverer for hydrophilic and hydrophobic agents, utilizing clinically osteoinductive nonpeptide drugs may be potential bioactive factors for bone tissue engineering because of low cost and facile availability. These drugs could be mainly divided into two groups: anabolic agents that promote bone regeneration and anticatabolic agents that inhibit bone absorption. For example, simvastatin (an anabolic drug)^[^ ^187^ ^]^ and bisphosphonate (an anticatabolic drug)^[^ ^188^ ^]^ have been utilized in bone tissue engineering when loaded on other biomaterials. Therefore, future studies should pay attention to the use of SPNHs to encapsule or conjugate osteoinductive nonpeptide drugs.

#### 3.2.3 Angiogenesis

Considering that bone is a highly vascularized tissue in which vasculature exerts critical effects in bone regeneration, remodeling, and maintaining, angiogenesis is highly needed to be considered when the passive diffusion for oxygen and nutrient transportation fails to occur during bone repair.^[^ ^189^ ^]^ Thus, angiogenic signals in biomaterials are beneficial for bone regeneration. A vast array of bioactive proteins exerts angiogenic effects, including vascular endothelial growth factor (VEGF),^[^ ^190^ ^]^ basic fibroblast growth factor (bFGF),^[^ ^191^ ^]^ and insulin‐like growth factors (IGFs),^[^ ^192^ ^]^ among which VEGF has been widely used as an angiogenic agent. For example, VEGF coupled with BMP‐2 was physically encapsuled into a PA hydrogel which was then loaded to collagen for mice critical cranial bone defects, and the results showed that VEGF‐incorporated scaffold promotes more new bone formation than scaffolds containing BMP‐2 only or without both VEGF and BMP‐2, which suggest that angiogenesis also exert indistinctive effects in bone regeneration.^[^ ^193^ ^]^

In addition to the physical adsorption of angiogenic factors, angiogenic motifs also show great promise to be covalently conjugated to SPNH matrix for bone tissue engineering, which can be obtained from bioactive molecules and natural ECM. KLTWQELYQLKYKGI, called QK or KLT, is a VEGF‐derived motif which has been tethered to several supramolecular peptides to stabilize its secondary structure and maintain its bioactivities.^[^ ^194^, ^195^ ^]^ And an IGF‐1‐derived motif (GYGSSSRRAPQT)^[^ ^196^ ^]^ and a heparin‐mimetic motif (K‐pbs)^[^ ^197^ ^]^ have been also tailored to supramolecular peptides to promote angiogenesis. Besides, ECM‐derived angiogenic motifs mainly include REDV and SVVYGLR: the former is a fibronectin‐derived tetrapeptide that selectively binds VECs by integrin *α*4*β*1 and induce angiogenesis;^[^ ^198^ ^]^ and the latter is a osteopontin‐derived motif which could specifically attach VECs and promote vascularization by *α*4*β*1and *α*9*β*1 integrins.^[^ ^199^ ^]^ While these bioactive motifs derived from bioactive molecules have been confirmed to promote angiogenesis when tethered to SPNH matrix, they have been rarely used for bone regeneration, but we believe that SPNHs containing both angiogenic motifs and osteogenic motifs may promote bone regeneration. Recently, Derkus et al.^[^ ^200^ ^]^ developed a multicomponent hydrogel comprising HA functionalized with tyramine and PAs containing adhesive motif (RGDS), osteogenic motif (DGEA), and angiogenic motifs (SVVYGLR). The hydrogel was then used for cocultures of ADSCs and VECs, and the results confirmed that the hybrid hydrogels could improve cell adhesion and promote osteogenic and angiogenic differentiation, ultimately forming vascularized bone‐like composites.^[^ ^200^ ^]^

#### 3.2.4 Neurogenesis

Analogous to vessels, nerve fibers are also widely distributed throughout the entire bone tissue, and they exert critical effects on bone metabolism, bone remodeling, bone cell functions, and nutrient exchange.^[^ ^201^ ^]^ Severe bone defects coupled with vascular and peripheral injuries may often result in nonunion of skeleton and other sequelae.^[^ ^202^ ^]^ Thus, neurogenesis should be considered for bone tissue engineering, which have been explored by encapsulating neurogenic factors into suitable biomaterials. Lei et al.^[^ ^203^ ^]^ fabricated injectable hydrogels embedded with osteogenic aspirin with or without neurogenic microRNA‐222, which were implanted to rat mandibular bone defects, and found that hydrogel containing both aspirin and microRNA‐222 promote more bone formation than hydrogels containing aspirin only or without both factors, which may be attributed to improved neurogenesis. Nerve growth factor (NGF) and brain‐derived neurotrophic factor (BDNF) are important bioactive factors for neural development and regeneration, and it has been shown that both NGF and BDNF indirectly or directly exert effects on osteogenesis.^[^ ^182^, ^202^, ^204^ ^]^ Considering that SPNHs are ideal carrier for bioactive factors, combing neurogenic factors and osteogenic factors into SPNHs may accelerate bone regeneration.

Various neurogenic motifs have been identified from neurogenic factors and natural ECM or by phage display screening, and functional SPNHs containing neurogenic motifs are permissive biomaterials for innervated bone regeneration, because these hydrogels have been widely exploited in nerve and brain tissue engineering.^[^ ^205^ ^]^ For neurogenic motifs derived from neurogenic molecules, BDNF‐mimetic peptides (such as RGIDKRHWNSQ and cyclic RKKADP)^[^ ^206^ ^]^ and neural‐cell‐adhesion‐molecule‐derived motifs (such as EVYVVAENQQGKSKA^[^ ^207^ ^]^ and SIDRVEPYSSTAQ^[^ ^208^ ^]^) have been developed for neurogenesis. For neurogenic motifs derived from natural ECM, laminin‐derived motifs, including IKVAV, tyrosine‐isoleucine‐glycine‐serine‐arginine (YIGSR), and arginine‐asparagine‐isoleucine‐alanine‐glutamate‐isoleucine‐isoleucine‐lysine‐asparate‐isoleucine (RNIAEIIKDI), have been used for nerve tissue engineering, and IKVAV and YIGSR were also confirmed to promote angiogenesis; thus, both motifs show promising prospects to form a neurovascularized network for bone regeneration.^[^ ^209^ ^]^ Furthermore, neurogenic motifs identified by identified by phage display screening include KLPGWSG^[^ ^210^ ^]^ and FAQRVPP.^[^ ^211^ ^]^ While these neurogenic motifs have been rarely tested in bone tissue engineering, we assert that SPNHs are attractive biomaterial to tether these neurogenic motifs for bone regeneration.

#### 3.2.5 Immune Regulation

In recent years, the role of immune system has been considered for bone tissue engineering. Various immune cells, such as neutrophils, macrophages, and T cells, exert effects in bone remodeling, among which macrophages are of high importance.^[^ ^212^ ^]^ After trauma, macrophages are mainly derived from circulating monocytes. Initially, macrophages show M1 phenotypes, which possess the ability to phagocytose dead cells and pathogens and promote inflammation, and then they transform to anti‐inflammatory M2 phenotypes, which could stimulate osteogenesis.^[^ ^213^ ^]^ Present studies mainly focused on inhibiting M1 macrophages and promoting M2 macrophages. For example, IL‐4 is a well‐characterized cytokine that could activate M2 phenotypes, and it was first loaded on graphene oxide (GO) coupled with BMP‐2 for controlled release.^[^ ^214^ ^]^ Then, both factors were embedded in carboxymethyl chitosan/PEG diacrylate hybrid hydrogels, and the results demonstrated that the hydrogel encapsulated with IL‐4 and BMP‐2 could induce M2 macrophage polarization and the osteogenic differentiation of MSCs in vitro, and reduce inflammation with enhanced bone neoformation in vivo.^[^ ^214^ ^]^ Besides, rosiglitazone is also an immunoregulator with the ability to promote M2 phenotypes, which has been used for bone regeneration.^[^ ^215^ ^]^ Therefore, these immune regulators could be directly or indirectly embedded in SPNFs for bone tissue engineering.

### 3.3 Additional Biochemical Functions

#### 3.3.1 Sterilization

Acute and chronic bone infections are difficult to treat because of bacterial colonies in the bone grooves, various factors released from the bacteria, bloodless materials, and acidic microenvironment.^[^ ^216^ ^]^ In clinic practice, osteomyelitis, an inflammation of bone or bone marrow that is generally caused by infection, is usually treated by completely clearing the lesion, followed by implanting an antibacterial material. Thus, the release of antibacterial signals should not be quick, as the remaining bacteria may disturb bone regeneration. SPNHs may be an ideal carrier for dual functions of sterilization and osteogenesis on the group where they exert dual roles as scaffolds and controlled release carriers. Yang et al.^[^ ^217^ ^]^ incorporated cationic antimicrobial peptides (AMPs) into RADA16 hydrogel, which could afford the controlled release of AMPs, inhibit the growth of *Staphylococcus aureus*, and promote bone regeneration at an osteomyelitis model of rabbit. In another study, ciprofloxacin was encapsulated into RADA16/calcium phosphate cement scaffold to prevent postoperative infection.^[^ ^218^ ^]^ Besides, some bioactive motifs could be directly conjugated to the backbone of supramolecular peptides for sterilization. As an example, the incorporation of consecutive arginine residues was confirmed to enhance the antimicrobial property of KLDL12 hydrogel, and KLD12—RR hydrogel simultaneously promoted more bone regeneration than KLD12 hydrogel.^[^ ^219^ ^]^

#### 3.3.2 Tumor Suppression

Large‐scale bone defects caused by tumor resection are also difficult to repair because of residual tumor cells, which may cause tumor relapse. Therefore, scaffolds with dual functions of tumor suppression and osteogenesis show great promise for tumor‐derived bone defects. Antitumor drugs with osteogenic properties are more suitable for bone regeneration than chemotherapeutic drugs that cause negative effects on MSCs. In one research, Mg particles were incorporated to multifunctional scaffolds, which allowed for tumor eradication under near‐infrared laser irradiation and osteogenesis induced by the released Mg ions.^[^ ^220^ ^]^ Another group introduced metformin, a common antidiabetic drug with multiple function including tumor suppression and osteogenesis promoting, to a controlled‐release scaffold, which could induce various functions, including antitumor and osteogenesis.^[^ ^221^ ^]^ Although SPNHs have been widely used for tumor excision, few studies used SPNHs as controlled‐release scaffolds for bone defects from tumor excision. Conjugating antitumor agents to the backbone of supramolecular peptides is also a promising strategy.

## 4 Mechanical Optimization for Physically Adapted Microenvironment

While SPNHs could fulfill structural emulation and biochemical functions, they may not be real competitors of traditional natural macromolecular hydrogels and synthetic polymer hydrogels because of their unsatisfying mechanical properties, which are mainly assumed to the noncovalent interactions. For MSCs, rigid matrix (25–40 kPa) is capable of stimulating osteogenic differentiation.^[^ ^238^ ^]^ However, the storage modulus (*G′*) of most SPNHs is 0.01–1 kPa.^[^ ^26^ ^]^ In addition, peptide hydrogels with *G′* ≈ 1 kPa may not be useful for osteogenesis because the elasticity of bone cells is over 30 kPa.^[^ ^239^ ^]^ Therefore, SPNHs are highly needed to provide physically adapted microenvironment for bone cells, and the stiff or rigid matrix could activate mechanotransduction‐related signaling pathways for bone regeneration.^[^ ^240^ ^]^

### 4.1 Gelation Conditions

**Figure 10:**
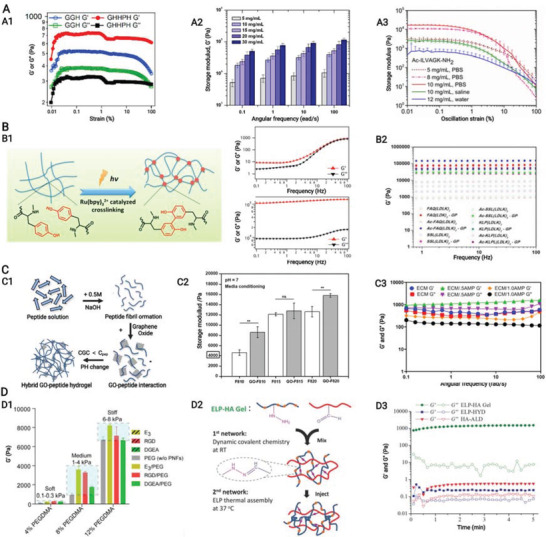
A–D) Mechanical optimization of supramolecular peptide nanofiber hydrogels, including modulation of gelation conditions, chemical cross‐links, physical cross‐links, and construction of interpenetrating network hydrogels. A1) Storage modulus could be improved by adding assembly sites (example of internal factors). Reproduced under the terms of the CC‐BY license.^[^ ^242^ ^]^ Copyright 2019, The authors, Published by MDPI. A2) Increasing gelation concentration (example of internal factors) could enhance the stiffness of peptide hydrogels. Reproduced with permission.^[^ ^83^ ^]^ Copyright 2011, Elsevier. A3) Peptide dissolving in different physiological solvents (example of internal factors) shows distinctive storage modulus. Reproduced with permission.^[^ ^243^ ^]^ Copyright 2015, American Chemical Society. B1) Chemical cross‐links by the ruthenium‐complex‐catalyzed conversion of tyrosine to dityrosine upon light irradiation could considerably improve the storage modulus of related aromatic peptide amphiphiles. Reproduced with permission.^[^ ^26^ ^]^ Copyright 2013, American Chemical Society. B2) Biocompatible genipin could be used to chemically linked supramolecular peptides containing primary amines, thus improving mechanical properties. Reproduced with permission.^[^ ^247^ ^]^ Copyright 2020, Elsevier. C1) Physical cross‐links by graphene oxide to enhance the connection between matrix nanofibers. C2) Modulating the storage modulus of SPNHs with different peptide concentrations by the introduction of graphene oxide. Reproduced under the terms of the CC‐BY license.^[^ ^248^ ^]^ Copyright 2019, The authors, Published by Elsevier. C3) Physical cross‐links by amorphous magnesium phosphates could improve the storage of FEFEFKFK peptide nanofiber hydrogels. Reproduced with permission.^[^ ^252^ ^]^ Copyright 2020, American Chemical Society. D1) Interpenetrating network hydrogels composed of peptide amphiphiles and poly(ethylene glycol) in different mixing ratios show improved storage modulus. Reproduced with permission.^[^ ^257^ ^]^ Copyright 2020, American Chemical Society. D2) Chemical cross‐links could also be introduced to an interpenetrating network hydrogel composed of elastin‐like polypeptide (ELP—HYD) and aldehyde‐modified hyaluronic acid (HA—ALD), which could fulfill two‐step cross‐linking. D3) At 25 °C, the mechanical properties of ELP—HYD, HA—ALD, and ELP—HA gels. Reproduced with permission.^[^ ^262^ ^]^ Copyright 2017, Wiley‐VCH. Created with BioRender.com.

Dozens of gelation conditions could modulate the mechanical properties of SPNHs, and they could be categorized into two groups: internal factors and external factors. Internal factors are related to the design of supramolecular peptides, which include the choice of amino acid, charge density, and distribution.^[^ ^241^ ^]^ Zeng et al.^[^ ^242^ ^]^ fabricated a peptide, glycine‐histidine‐histidine‐proline‐histidine (GHHPH), which showed more histidine—Zn^2+^ binding than glycine‐glycine‐histidine (GGH); thus, its mechanical property was improved (**Figure** **10**A1). External factors include gel concentration, solvent, ion strength, pH, and temperature. While ion strength, pH, and temperature could influence the stiffness of SPNHs, they are highly supposed to be limited to physiological conditions. Related orchestrations by these parameters may not be suitable for bone tissue engineering. Therefore, the mechanical properties of SPNHs are mainly modulated by gel concentration and optimized physiological solvent. The storage modulus could be generally enhanced by improving the gel concentration for most SPNHs. As an example, the storage modulus of Ac—LD6 or Ac—ID3 increased with the increase in concentration (Figure 10A2).^[^ ^83^ ^]^ However, the porosity of SPNHs may be spoiled by high concentration. Therefore, a balance between gel concentration and matrix porosity should be maintained in practical application. Besides, supramolecular peptides dissolved in different physiological solvents may show different *G′* values, which may be related to specific ions. The *G′* of a pentapeptide (Ac—ILVAGK—NH_2_) dissolved in PBS was extensively higher than that of a peptide dissolved in normal saline (Figure 10A3).^[^ ^243^ ^]^

### 4.2 Chemical Cross‐Links

Chemical cross‐links are strategies to improve mechanical properties by establishing cross‐linking between nanofibers. It could be realized by specific amino acids of supramolecular peptide or additional cross‐linkers. Ding et al.^[^ ^26^ ^]^ fabricated two APAs containing tyrosine (Fmoc—FFY and Fmoc—FFGGGY), and both peptides could form nanofiber hydrogels, which were then cross‐linked by the conversion of tyrosine to dityrosine in the presence of ruthenium complex and light irradiation. The *G′* was considerably improved by 10^4^‐fold to ≈100 kPa (Figure 10B1).^[^ ^26^ ^]^ Besides, Pugliese et al.^[^ ^244^ ^]^ utilized sulfo‐SMCC (an aminie‐to‐sulfhydryl corss‐linker) to chemically interlink CK peptide (Ac—CGGLKLKLKLKLKLKGGC—CONH_2_). Both ends of sulfo‐SMCC are *N*‐hydroxysuccinimide ester and maleimide groups, which could react with the amino and sulfhydryl groups of CK peptide to form stable covalent bonds.^[^ ^244^ ^]^ The cross‐linked SPNH showed an extensively improved *G′* of 170 kPa in the presence of 10 × 10^−3^ m sulfo‐SMCC, while the un‐cross‐linked SPNH displayed a soft *G′* of 5 kPa.^[^ ^244^ ^]^ Another group established a dual enzymatic polymerization: a supramolecular peptide (Nap—FFK—acrylic acid) was first assembled into SPNH (gel I), and then some agents for polymerization were added to form gel II, including glucose oxidase, glucose, horseradish peroxidase (HRP), acetyl acetone (AcAc), and PEG methacrylate.^[^ ^245^ ^]^ The mechanism of secondary gelation is that glucose was oxidated to gluconic acid by Gox, and O_2_ was reduced to H_2_O_2_ by the flavin adenine dinucleotide cofactor.^[^ ^245^ ^]^ Subsequently, AcAc, under the catalyzation of HRP, reacted with H_2_O_2_ and formed AcAc radicals, which induced the chemical cross‐linking of PRGMA and the supramolecular peptides.^[^ ^245^ ^]^ Ultimately, the mechanical properties of gel II were remarkably improved compared with those of gel I.^[^ ^245^ ^]^ However, the aforementioned strategies need specific peptide sequence and potentially toxic reagents, thus limiting their further applications. Genipin is a natural cross‐linker extracted from *Gardenia jasminoides*, and it could react with primary amines of proteins and peptides.^[^ ^246^ ^]^ It is biocompatible, with far less cytotoxicity (5000–10 000 times) than glutaraldehyde.^[^ ^236^ ^]^ Pugliese et al.^[^ ^247^ ^]^ fabricated a series of lysine‐containing supramolecular peptides that could assemble into nanofiber hydrogels and be further cross‐linked by genipin.^[^ ^247^ ^]^ The chemical cross‐linking considerably improved the *G′* by 50–100 times, but it did not spoil the assembling nanostructures (Figure 10B2).^[^ ^247^ ^]^ It did not even disturb the biochemical functions, because no bioactive motifs were involved.^[^ ^247^ ^]^ Therefore, functional lysine‐containing supramolecular peptides coupled with genipin demonstrate great promise for bone tissue engineering.

### 4.3 Physical Cross‐Links

Physical cross‐links could be used to modulate mechanical properties, and it needs the introduction of specific agents (either inorganic or organic) to interact with nanofibers. Ligorio et al.^[^ ^248^ ^]^ used GO as a physical cross‐linker to enhance a SPNH: short thin fibrils formed by FEFKFEFK were initially deposited on the surface of GO flakes, and then GO physically cross‐linked matrix nanofiber after these fibrils were induced to form SPNHs (Figure 10C2).^[^ ^248^ ^]^ At physiological pH, only hydrophobic interaction controlled the physical cross‐links (namely, interfacial adhesion) between GO and peptide nanofibers, and it was found that the *G′* of SPNH with 10 mg mL^−1^ peptide concentration (F810) and SPNH with 20 mg mL^−1^ peptide concentration (F820) were dramatically enhanced by GO (Figure 10C2).^[^ ^248^ ^]^ However, GO could not be degraded in vivo, thereby limiting its further application in regenerative medicine. Thus, biodegradable HAP, which is an intrinsic element of natural bone, is more preferred than GO. Anderson et al.^[^ ^249^ ^]^ encapsulated HAP nanoparticles in RGDS‐tethered PA hydrogel, and found that the *G′*/loss modulus (*Gʺ*) value was improved form 4.085 ± 0.81 to 8.494 ± 0.71 and 4.594 ± 0.89 by 50% and 66.7% HAP, respectively, but 33% HAP lowered the value to 4.541 ± 0.72. The RGDS‐tethered PA hydrogel at 50% HAP could induce osteogenic differentiation of MSCs in vitro and promote bone regeneration at critical‐size femoral defects in rats.^[^ ^249^ ^]^ And further study showed that SPNH containing 66% HAP showed better osteogenic effects than SPNH containing 50% HAP.^[^ ^250^ ^]^ However, the direct encapsulation of HAP to SPNHs is limited because insoluble HAP generally causes aggregation, which hinders their potential utility as bone substitutes for bone tissue engineering. So, it has been reported that a phosphate group was introduced to a supramolecular peptide to enhance the interaction between the hydrogel matrix and HAP, which could overcome HAP aggregation and even dramatically improve the *G′* of the SPNH at relatively low concentration.^[^ ^251^ ^]^ Besides, highly soluble bioactive amorphous magnesium phosphate (AMP) was encapsulated into FEFKFEFK hydrogel; it could not only improve *G′* but also promote osteogenesis (Figure 10C3).^[^ ^252^ ^]^ Therefore, modifying supramolecular peptides or using highly soluble and degradable inorganic particles are ideal strategies to optimize the direct introduction of HAP to improve the mechanical properties of SPNHs.

In addition to inorganic particles, organic macromolecules could be used to improve the mechanical properties of SPNHs, among which proteins and polysaccharides have been used to coassemble with supramolecular peptides. And these macromolecules are characterized by that they cannot form fibrous hydrogels in isolation. Javid et al.^[^ ^253^ ^]^ incorporated dilute protein clusters at low concentrations to SPNHs, and found that both bovine serum albumin and *β*‐lactoglobulin at low concentration of 0.03 wt% could improve the *G′* of Fmoc—YL and Fmoc—VL hydrogel by peptide–protein interaction and confinement by protein clusters. But the improvement in mechanical properties was attenuated with the increase of protein concentration, which may be attributed to the interpretation that high concentrations improve the peptide–protein interactions and lead to thick fibers by protein coating.^[^ ^253^ ^]^ Also, another group utilized dextran, a highly hydrophilic polysaccharide, to modulate the mechanical properties and found that low concentration dextran improves the *G′* of a SPNH, while high concentration dextran decreases the *G′*.^[^ ^254^ ^]^ Therefore, low concentration of macromolecules may improve the mechanical properties of SPNHs. To enhance the interaction between peptide nanofibers and macromolecules, highly charged proteins or polysaccharides could be chosen to enhance the effects of binding and tempting. Xing et al.^[^ ^255^ ^]^ used a cationic polypeptide (poly‐l‐lysine, PLL) with a thiol group to improve the mechanical properties of anionic Fmoc—FF hydrogel at low concentration. The electrostatic interaction between PLL and hydrogel nanofiber enhanced the mechanical properties and allowed for self‐healing and shear thinning, and the interaction could also be further enhanced by disulfide bonds.^[^ ^255^ ^]^ Furthermore, bioactive macromolecules with multiple functions may be the future direction for biomedical applications. Fucoidan, a polyanionic polysaccharide containing vegetal fucose, could bind and interact with heparin‐binding bioactive factors to improve their bioactivity and promote wound healing, so it was encapsuled into a SPNH.^[^ ^256^ ^]^ The results showed fucoidan at low concentrations is presented on the surface of peptide nanofibers to form thicker bundles and that coassembling fucoidan and supramolecular peptide dramatically improves the *G′* of a SPNH from 10 to 1500 Pa by the effects of binding and tempting.^[^ ^256^ ^]^ Therefore, tuning the mechanical properties of SPNHs by bioactive macromolecules is urgent to explore in the field of bone tissue engineering.

### 4.4 Interpenetrating Network (IPN)

IPN involves constructing multinetwork hydrogels which are characterized by fully or partially interfaced networks.^[^ ^214^ ^]^ PEG is one of the most commonly used biomaterials that could provide precise mechanical control with good resistance to proteolysis. Goktas et al.^[^ ^257^ ^]^ successfully fabricated a mechanically tunable IPN hydrogel by blending a PA hydrogel and a chemically cross‐linked PEG network. The former allowed for incorporating bioactive motifs to hybrid hydrogel, while the latter could effectively modulate the mechanical properties by concentration changes.^[^ ^257^ ^]^ With the increase of the PEG concentration from 4%, 8% to 12%, the *G′* of the IPN hydrogel increases from 0.1–0.3, 1–4 to 6–8 kPa (Figure 10A1).^[^ ^257^ ^]^ Besides, other hydrogelating macromolecules that have been reported to construct IPN hydrogels to improve the mechanical properties of SPNHs alone include agarose,^[^ ^258^ ^]^ guar gum,^[^ ^259^ ^]^ alginate,^[^ ^260^ ^]^ and HA.^[^ ^261^ ^]^ Furthermore, the multinetwork within IPN hydrogels could be covalently connected to one another. Wang et al.^[^ ^262^ ^]^ separately designed two biomaterials: hydrazine‐modified ELP (ELP—HYD) and aldehyde‐modified HA (HA—ALD). After both biomaterials were mixed, ELP was tethered to HA by covalent hydrazone bonds, which induced the formation of initial hydrogel.^[^ ^262^ ^]^ And when the temperature was above the Tt of ELP (25.6 °C), the physical assembly of ELP allowed for a secondary cross‐linking to form a reinforced hydrogel (Figure 10D2).^[^ ^262^ ^]^ At 25 °C that is slightly below Tt, the results of mechanical properties showed that the *G′* of both ELP—HYD and HA—ALD were below 1 Pa, but the *G′* of ELP—HA hydrogel which contained covalently connected IPN reached ≈1000 Pa, which indicates that the formation of covalently linked IPN could dramatically improve the mechanical properties of the SPNH (Figure 10D3).^[^ ^262^ ^]^ Then the ELP—HA hydrogels (4, 1 wt%) enhanced by the secondary ELP assembly were used to 3D culture MSCs, which show that the enhanced hydrogel supported cell culture for 3 weeks, enabled MSCs to maintain viability and osteogenic differentiation.^[^ ^262^ ^]^

While IPN is a promising strategy to enhance the mechanical properties of SPNHs, it should be carefully used for bone tissue engineering because the introduction of another network to SPNHs may change the matrix density and porosity, which may abrogate cell behaviors. So, it is highly recommended to optimize the mixing ratio of supramolecular peptides and other hydrogelating molecules, and enzyme‐clearable IPN hydrogels may be another method to overcome the challenge because enzyme digestion sites within hydrogel matrix may allow for cell infiltration and execute other cell behaviors.

## 5 Comprehensive Applications of SPNHs for Bone Tissue Engineering

In clinic practice, bone defects may occur at any nonbearing or bearing areas. For nonbearing bone defects, injectable hydrogels may be ideal therapeutic strategies because they allow for minimally invasive intervention and adaptive matching of irregular defects.^[^ ^263^ ^]^ However, for bearing bone defects, composite nanoscaffolds (porous scaffolds interpenetrated with SPNHs) are superior. Furthermore, bioprinting could be used to construct complicated bone grafts, which could realize targeted or oriented distribution of multiple cells or bioactive motifs or factors. Bioreactors that generate mechanical stimuli could also be used to fabricate tissue‐engineered bone grafts in vitro. Transplanting these artificial bone grafts has attracted great attention.

### 5.1 Injectable Hydrogels

**Figure 11:**
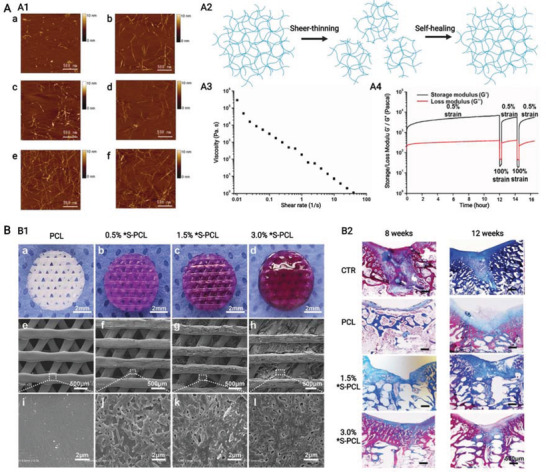
A,B) Comprehensive applications of supramolecular peptide nanofiber hydrogels in the form of injectable hydrogels or composite nanoscaffolds. A1) RADA16‐I nanofiber hydrogel could be broken by sonication and then undergo sol–gel transition over time: 1 min (a), 4 min (b), 16 min (c), 32 min (d), 1 h (e), and 24 h (f) after sonication. Reproduced with permission.^[^ ^28^ ^]^ Copyright 2005, National Academy of Sciences. A2) Schematic of mechanoresponsive injectable hydrogels with thixotropic property (shear thinning and self‐healing). A3‐A4) Interpenetrating network hydrogel composed of Fmoc—FF and alginate show thixotropic property. Reproduced under the terms of the CC‐BY license.^[^ ^260^ ^]^ Copyright 2019, The authors, Published by MDPI. B1) Morphology and scanning electron microscopic images of composite nanoscaffolds composed of printed polycaprolactone scaffolds and FEFKFEKF nanofiber hydrogels. B2) Microfracture healing results revealed by Masson staining in CTR (microfracture without grafts), PCL alone, 1.5% FEFKFEKFK‐coated PCL, and 3.0% FEFKFEFK‐coated PCL groups at 8 and 12 weeks. Reproduced with permission.^[^ ^47^ ^]^ Copyright 2019, Wiley‐VCH. Created with BioRender.com.

Injectable hydrogels refer to those that could be delivered to bone defects by catheters or syringes. The property of sol–gel transition is the basic requirement for injectable hydrogels, that is, hydrogels are delivered in liquid‐like state (sol) but then transferred to solid‐like state (gel) triggered by various factors, including temperature, ion strength, pH, light, and external stimuli. Ionic‐complementary peptides are common injectable hydrogels that could be transferred to sol state after sonication, and nanofibers could recover in ≈2 h (**Figure** **11**A1).^[^ ^28^, ^31^ ^]^ However, there are two main drawbacks hindering their applications in regenerative medicine. On the one hand, most ionic‐complementary peptides may briefly lower the pH upon immersion in aqueous solution, which damages cells and host tissues upon direct exposure. So, incorporating 10% sucrose to the hydrogel system is highly suggested because the isosmotic sucrose could protect cells and host tissues from the low pH microenvironment until the hydrogel system is neutralized to physiological pH.^[^ ^123^, ^264^ ^]^ Besides, modifying ionic‐complementary peptides by oppositely charged functional motifs is another emerging approach to optimize the low pH microenvironment. Sun et al.^[^ ^205b^ ^]^ fabricated a SPNH assembled by oppositely charged RADA16—DGDRGDS and RADA16—RIKVAV, which could effectively avoid the death of seeded cells compared with RADA16 hydrogel. One the other hand, ionic‐complementary peptides may not be suitable for encapsulated cells or bioactive factors because the gelation is induced by stimuli. So, mechanoresponsive injectable hydrogels are preferrable, which are charactered by thixotropic properties, namely, shear thinning and self‐healing. Upon strain loading or shear stress, the hydrogel is broken into small domain in sol state, which may gradually recover to gel state when external mechanical loading is depleted (Figure 11A2). *β*‐Hairpin peptides are common examples of thixotropic hydrogels. In recent years, some IPN hydrogels have shown mechanoresponsive property. For example, an IPN hydrogel composed of Fmoc—FF and alginate extensively improved the *G′* of Fmoc—FF hydrogel (from 1.8 to 10 kPa).^[^ ^260^ ^]^ With the increase in shear rate (0.01–100 s^−1^), the viscosity of the IPN hydrogel gradually decreased, thus showing the property of shear thinning (Figure 11A3).^[^ ^260^ ^]^ The mechanical strength of the IPN hydrogel could restore the large‐amplitude oscillatory breakdown (100% strain), thus showing the property of self‐assembling (Figure 11A4).^[^ ^260^ ^]^ When the IPN hydrogel was used to culture MC3T3‐E1 preosteoblasts by osteogenic induction medium, it was found that the IPN hydrogel supported matrix mineralization and cell osteogenic differentiation, as confirmed by Alizarin red staining, enhanced ALP activity, and improved mRNA expression of osteogenic genes including ALP, RUNX2, osteocalcin, BMP‐2, and type I collagen.^[^ ^260^ ^]^ And the osteogenic ability should be further tested in models of critical bone defect.

### 5.2 Composite Nanoscaffolds

Composite nanoscaffolds are fabricated by infiltrating SPNHs into porous scaffolds. They show hierarchical structure with macroscale patterns and nanoscale topography, and they are more analogous to the complex bone tissue. Endogenous cell recruitment and migration may not be affected because of the weak intermolecular noncovalent interactions. Porous scaffolds could effectively preserve SPNHs from proteolysis, while SPNHs could provide an artificial ECM to enhance biochemical functions, such as cell adhesion. SPNHs have been currently loaded into a plethora of porous scaffolds for bone repair. Examples are the upgrade from undegradable metal scaffolds, such as titanium foam,^[^ ^265^ ^]^ to biodegradable scaffolds, such as collagen sponge^[^ ^154^, ^266^ ^]^ and demineralized bone matrix^[^ ^267^ ^]^ and next to hybrid biphasic scaffolds, such as HAP/chitosan^[^ ^268^ ^]^ and HAP/polyamide 66.^[^ ^269^ ^]^ In recent years, 3D printed scaffolds are thriving because additive manufacturing techniques allow for the potential to fabricate patient‐specific products that match the shape and size of patients’ bone defects by computer‐assisted technologies.^[^ ^270^ ^]^ Li et al.^[^ ^47^ ^]^ fabricated composite nanoscaffolds by combing SPNHs assembled by FEFEFKFK in different concentrations and 3D printed polycaprolactone (PCL) scaffold (Figure 11B1), and found that composite nanoscaffolds showed enhanced hydrophilicity and mimetic ECM‐like microenvironment when compared with PCL scaffold alone. The composite nanoscaffolds containing 1.5% or 3% SPNHs dramatically enhanced the osteogenic differentiation of MSCs in vitro, and promoted bone regeneration after 8 and 12 weeks in vivo (Figure 11B2).^[^ ^47^ ^]^ Moreover, to further simulate the complexity of natural bone, future studies should focus on osteoinductive scaffolds, such as ion (Sr^2+^, Mg^2+^, and/or Zn^2+^)‐doped scaffolds,^[^ ^271^ ^]^ because osteoinductive scaffolds could also provide osteogenic stimuli to enhance bone healing.

### 5.3 Bioprinting

**Figure 12:**
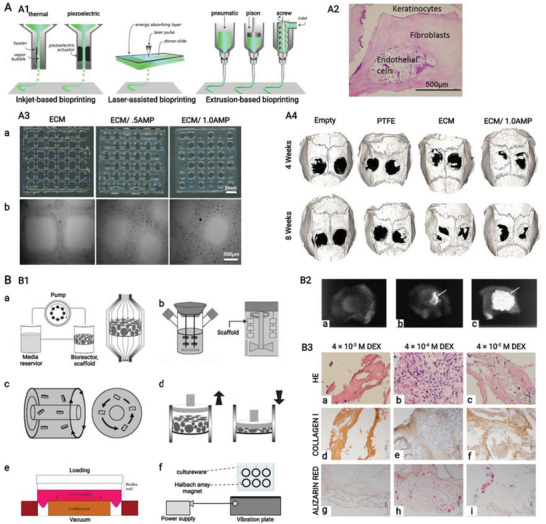
A,B) Comprehensive applications of supramolecular peptide nanofiber hydrogels by bioprinting or bioreactors. A1) Widely used bioprinting techniques. Reproduced with permission.^[^ ^273^ ^]^ Copyright 2013, Wiley‐VCH. A2) Multiple cells could be encapsuled to specific domains within hydrogels to construct artificial‐tissue‐like structure by bioprinting. Reproduced with permission.^[^ ^243^ ^]^ Copyright 2015, American Chemical Society. A3) Morphology and optical microscopic images of bioprinting FEFKFEFK nanofiber hydrogels containing amorphous magnesium phosphates (AMPs), and A4) critical bone healing results revealed by microcomputed tomography (micro‐CT) in empty (bone defects without grafts), ECM (FEFKFEFK nanofiber hydrogel alone), ECM/1.0 AMP (FEFKFEFK nanofiber hydrogel containing 1% AMP) at 4 and 8 weeks. Reproduced with permission.^[^ ^252^ ^]^ Copyright 2020, American Chemical Society. B1) Various bioreactors for the construction of tissue‐engineered bone grafts: perfusion bioreactor (a); spinner flask bioreactor (b); rotating wall vessel bioreactor (c); and compression bioreactor (d). Reproduced under the terms of the CC‐BY license.^[^ ^277a^ ^]^ Copyright 2021, The authors, Published by MDPI. Stretching bioreactor (e). Reproduced under the terms of the CC‐BY license.^[^ ^277b^ ^]^ Copyright 2020, The authors, Published by Springer Nature. Nanovibration bioreactor (f). Reproduced under the terms of the CC‐BY license.^[^ ^277c^ ^]^ Copyright 2019, The authors, Published by Springer Nature. B2) Ectopic bone formation in vivo revealed by soft X‐ray: tissue‐engineered bone graft by a perfusion bioreactor to culture a composite nanoscaffold composed of peptide amphiphile nanofiber hydrogel and collagen (a), composite nanoscaffold cultured in perfusion bioreactor by static culture methods (b), composite nanoscaffold in normal medium by static culture method (c). Reproduced with permission.^[^ ^278^ ^]^ Copyright 2006, Elsevier. B3) Histological results of a tissue‐engineered bone tissue by three‐modality approach (mesenchymal stem cells, dexamethasone into RADA16‐I nanofiber hydrogel, and perfusion bioreactor). Reproduced under the terms of the CC‐BY license.^[^ ^279^ ^]^ Copyright 2019, The authors, Published by MDPI. Created with BioRender.com.

Bioprinting, another distinctive branch of additive manufacturing techniques, allows for fabricating complicated tissue by precise deposition of biomaterials, living cells, and/or bioactive motifs/factors in a layer‐by‐layer model.^[^ ^272^ ^]^ The three major approaches for bioprinting are inkjet‐based bioprinting, laser‐assisted bioprinting, and extrusion‐based bioprinting (**Figure** **12**A1). Among them, inject‐based printing is the most popular because it yields scaffolds with elaborate microenvironment and enables accurate placement of living cells and/or bioactive motifs/factors.^[^ ^273^ ^]^ The major challenge of inject bioprinting, however, is the devoid of suitable bioprinting materials. SPNHs have been recently recognized as promising inject‐based bioinks for tissue engineering because of their excellent properties, including shear thinning, self‐healing, shape memory, stimuli gelation, facile functionalization, and tunable mechanical strength.^[^ ^16^, ^274^ ^]^ However, various SPNHs must still be optimized as follows to maintain the balance between physical properties and biological performance: 1) fast gelation after bioprinting to encapsule living cells and bioactive factors, 2) low viscosity and stiffness for cell migrations, nutrient transmission, or high stiffness for structural integrity and geometrical fidelity.^[^ ^20a^ ^]^

In 2015, an ultrashort peptide (Ac—ILVAGK—NH_2_) was first utilized as a bioprinting ink, and it could instantly assemble into rigid nanofiber hydrogels with storage moduli up to 40 kPa when triggered by PBS.^[^ ^243^ ^]^ The bioprinting hydrogel was obtained by sequential deposition of peptide stocking solution and PBS containing living cells and/or bioactive factors, which could support 3D cell culture and differentiation.^[^ ^243^ ^]^ Multiple cells could be encapsuled to specific domains within hydrogels to construct artificial‐tissue‐like structure by sequential deposition SPNHs seeded with different cells (Figure 12A2).^[^ ^243^ ^]^ However, complementary supramolecular peptides may be superior to such stimuli responsive self‐assembling peptides when used as bioinks, because the shift of solution condition could be avoided, which may be more compatible for delivering living cells and bioactive motifs/factors. Jian et al.^[^ ^275^ ^]^ designed two mutually attractive supramolecular peptides, Fmoc—YD and Fmoc—YK, which could assemble into stable hydrogel by facile mix at homogeneous conditions. Two peptides were sequentially deposited in a layer‐by‐layer fashion, ultimately forming a robust hydrogel with storage moduli up to 42 kPa when the mix ratio was 1:1.^[^ ^275^ ^]^ So, coassembling SPNHs may be more biocompatible than stimuli‐induced SPNHs for bone tissue engineering, which needs further attention. Since constructing SPNHs by bioprinting is a new technique in recent years, bioprinted SPNHs have not been widely tested for osteogenesis and bone regeneration, and specific bioprinting methods and parameters are also needed to establish.

Constructing hybrid supramolecular peptide bioinks with excellent mechanical properties is reasonable. Natural proteins (such as keratin and gelatin) and inorganic particles (such as AMPs) were used to fabricate hybrid bioinks.^[^ ^252^, ^276^ ^]^ For instance, Dubey et al.^[^ ^252^ ^]^ introduced AMPs to FEFEFKFK stock solution to obtain a biphasic bioink (Figure 12A3), and that the printed hydrogel containing AMPs in vitro supported DPSC viability and induced osteogenic differentiation and matrix mineralization.^[^ ^252^ ^]^ The cell‐free hydrogels with or without AMPs were also implanted to rat calvarial defects, and the AMP‐distributed hydrogels showed more bone volume and improved bone density compared with the hydrogel without AMPs (Figure 12A4).^[^ ^252^ ^]^ Therefore, bioprinting SPNHs containing osteogenic signaling has a great promise for clinical patient‐specific bone repair.

### 5.4 Bioreactors

In addition to bioprinting, complicated artificial bone substitutes could be also obtained by bioreactors in vitro, which are generally called tissue engineered bone grafts (TEBGs). TEBGs are generally assumed as the secondary autogenous bone grafts, which could be implanted to promote bone repair, especially for bone defects caused by the excision of infection or tumor, and osteoporotic bone defects which are caused by fractures in osteoporosis patients, because microenvironments in these cases are not suitable for bone regeneration. Multiple bioreactors are shown in Figure 12B1.^[^ ^277^ ^]^ The strategies using bioreactors need ideal biomaterials for 3D culture of MSCs, and SPNHs are suitable matrix for this requisite. Kobayashi and co‐workers^[^ ^278^ ^]^ used a perfusion bioreactor to culture MSCs in a composite nanoscaffold composed of a collagen sponge and a PA hydrogel. They verified that the osteogenic differentiation of MSCs was greatly influenced by dynamic perfusion culture compared with static culture, as confirmed by that dynamic culture of MSCs in the perfusion bioreactor showed higher ALP activity and osteocalcin content than static culture.^[^ ^278^ ^]^ Then, hybrid scaffolds were implanted to the back subcutis of rats, and grafts derived from perfusion bioreactor extensively induced homogeneous ectopic bone regeneration compared with those from static culture (Figure 12B2).^[^ ^278^ ^]^ Another group encapsuled MSCs and dexamethasone into RADA16 hydrogel and used a perfusion bioreactor to provide shear stress to further stimulate osteogenic differentiation, and found that 4 × 10^−4^ m‐contained RADA16 hydrogel produces higher mRNA expression of osteogenic genes including type I collagen and osteocalcin when induced in the perfusion bioreactor.^[^ ^279^ ^]^ And histomorphometric analysis by hematoxylin–eosin staining, type I collagen immunohistochemistry staining, and Alizarin Red staining also confirmed the osteogenic differentiation of MSCs and the formation of mineralized bone matrix (Figure 12B3).^[^ ^279^ ^]^ However, in vivo studies using bone defect models are still needed to test these TEBGs based on SPNHs. And further studies could focus on other bioreactors other than perfusion bioreactors by SPNHs and achieve preferable loading regiment.

## 6 Conclusions and Outlook

The emerging SPNHs showed great promise to mimic the complexity of multihierarchical bone from three aspects: structural features, biochemical functions, and mechanical properties. For structural design, a plethora of hydrogelating supramolecular peptides with distinctive building blocks was systematically reviewed, and these peptides could assemble into ECM‐like scaffolds. For biochemical functionalization, two methodologies could be executed: covalent conjugation of bioactive motifs and physical encapsulation of bioactive factors. Bioactive motifs or factors could be used to endow SPNHs with basic functions for general tissue engineering (cell adhesion, cell recruitment, and matrix degradation), improved functions for bone tissue engineering (osteogenesis, angiogenesis, neurogenesis, and immune regulation), and additional functions for specific treatments (sterilization and tumor suppression). For mechanical optimization, four strategies were developed to improve the mechanical properties of SPNHs, including optimizing gelation conditions, chemical cross‐links, physical cross‐links, and IPNs. Furthermore, SPNHs are considered to be utilized in the form of injectable hydrogels or composite nanoscaffolds. Bioprinting and bioreactors could be used to fabricate engineered bone grafts, which could be then transplanted for bone repair.

While various basic supramolecular peptides based on distinctive building blocks were developed, novel basic peptides should also be fabricated by rational design, which should meet the following criteria: 1) improved biocompatibility with no toxicity; 2) stability at neutral environment; 3) tunable mechanical properties; 4) facile synthesis and low cost; 5) feasible functionalization; and 6) injectability (sol–gel transition) with thixotropic property (shear thinning and self‐healing). In addition to embedding or conjugating BMPs or related peptides, future studies should focus on other osteoinductive factors or motifs, especially PTH, related peptides, and EVs. Neurogenesis and immune regulation during bone regeneration should also receive attention. Furthermore, ideal mechanical‐adopted microenvironment for bone regeneration and mechanotransduction should be explored, which may guide future studies in effectively modulating the mechanical properties of SPNHs. Optimal strategies to control the mechanical properties should be also established. At present, bioactive motifs or factors are randomly distributed to the matrix of SPNHs. Thus, bioprinting provides an ideal strategy for bioactive signaling to target delivery, and related bone grafts should be tested in models of bone defects or multitissue defects. Besides, preconstruction in vitro by different bioreactors shows great promise for bone repair, especially for those with microenvironment unsuitable for bone regeneration. However, related techniques and optimized parameters should be established. Tissue‐engineered bone grafts should also be further transplanted in vivo.

Even though SPNHs for bone regeneration were tested in animal models, future studies should focus on clinical evidence. Related sterilization strategies for SPNHs should also be developed for translational medicine. SPNHs may surpass natural biomaterial hydrogels and synthetic polymer hydrogels in the future when the challenges are solved, and they may be ultimately used for clinical surgical bone repair.

## Conflict of Interest

The authors declare no conflict of interest.
